# Natriuretic peptides and C‐reactive protein in in heart failure and malnutrition: a systematic review and meta‐analysis

**DOI:** 10.1002/ehf2.14851

**Published:** 2024-06-08

**Authors:** Konstantinos Prokopidis, Krzysztof Irlik, Hironori Ishiguchi, Willemina Rietsema, Gregory Y.H. Lip, Rajiv Sankaranarayanan, Masoud Isanejad, Katarzyna Nabrdalik

**Affiliations:** ^1^ Department of Musculoskeletal and Ageing Science, Institute of Life Course and Medical Sciences University of Liverpool Liverpool UK; ^2^ Liverpool Centre for Cardiovascular Science at University of Liverpool Liverpool John Moores University and Liverpool Heart & Chest Hospital Liverpool UK; ^3^ Students' Scientific Association by the Department of Internal Medicine, Diabetology and Nephrology in Zabrze, Faculty of Medical Sciences in Zabrze Medical University of Silesia Katowice Poland; ^4^ Division of Cardiology, Department of Medicine and Clinical Science Yamaguchi University Graduate School of Medicine Ube Japan; ^5^ Oxford Health NHS Foundation Trust Oxford UK; ^6^ Danish Center for Health Services Research, Department of Clinical Medicine Aalborg University Aalborg Denmark; ^7^ Liverpool University Hospitals NHS Foundation Trust Liverpool UK; ^8^ National Institute for Health and Care Research London UK; ^9^ Department of Internal Medicine, Diabetology and Nephrology, Faculty of Medical Sciences in Zabrze Medical University of Silesia Katowice Poland

**Keywords:** heart failure, malnutrition, BNP, NT‐proBNP, CRP

## Abstract

**Background:**

Heart failure (HF) and malnutrition exhibit overlapping risk factors, characterized by increased levels of natriuretic peptides and an inflammatory profile. The aim of this study was to compare the differences in plasma brain natriuretic peptide (BNP), N‐terminal‐pro B‐type natriuretic peptide (NT‐proBNP), and C‐reactive protein (CRP) in patients with HF and malnutrition versus normal nutrition.

**Methods:**

From inception until July 2023, the databases, PubMed, Scopus, Web of Science, and Cochrane Library were searched. To examine the association among malnutrition [controlling nutritional status (CONUT) score ≥2; Geriatric Nutritional Risk Index (GNRI) score <92] with BNP, NT‐proBNP and CRP in patients with HF, a meta‐analysis using a random‐effects model was conducted (CRD42023445076).

**Results:**

A significant association of GNRI with increased levels of BNP were demonstrated [mean difference (MD): 204.99, 95% confidence interval (CI) (101.02, 308.96, *I* ^2^ = 88%, *P* < 0.01)], albeit no statistically significant findings were shown using CONUT [MD: 158.51, 95% CI (−1.78 to 318.79, *I* ^2^ = 92%, *P* = 0.05)]. GNRI [MD: 1885.14, 95% CI (1428.76–2341.52, *I* ^2^ = 0%, *P* < 0.01)] and CONUT [MD: 1160.05, 95% CI (701.04–1619.07, *I* ^2^ = 0%, *P* < 0.01)] were associated with significantly higher levels of NT‐proBNP. Patients with normal GNRI scores had significantly lower levels of CRP [MD: 0.50, 95% CI (0.12–0.88, *I* ^2^ = 87%, *P* = 0.01)] whereas significantly higher levels of CRP were observed in those with higher CONUT [MD: 0.40, 95% CI (0.08–0.72, *I* ^2^ = 88%, *P* = 0.01)]. Employing meta‐regression, age was deemed a potential moderator between CRP and GNRI.

**Conclusions:**

Normal nutrition scores in patients with HF are linked to lower BNP, NT‐proBNP, and CRP levels compared with malnourished counterparts. Despite the significant link between CRP and malnutrition, their relationship may be influenced in older groups considering the sensitivity of GNRI due to ageing factors.

## Introduction

Malnutrition is a widespread challenge, encompassing both undernutrition and overnutrition. The current health emergency affects all segments of the population, particularly individuals in older age, those with low socioeconomic status, and those suffering from chronic or acute conditions.^1^ Malnutrition in patients on hospital admission is highly prevalent, ranging from 20% to 50%,^2^, ^3^, ^4^ a phenomenon that depends on the type of patient and the assessment tool for malnutrition.

One clinical group susceptible to malnutrition are patients with heart failure (HF), for which malnutrition may be prevalent up to 69% of patients^5^ and is a prognostic factor of hospitalization,^6^ incident mortality,^7^ and major cardiovascular events.^8^, ^9^ Although there are multiple malnutrition tools, the Controlling Nutritional Status (CONUT) score and the Geriatric Nutritional Risk Index (GNRI) are some of the most adopted tools in patients with HF. The GNRI is calculated using the formula: 1.489 × serum albumin (g/L) + 41.7 × (body weight in kg/ideal body weight which is calculated suing the equation: 22 × height^2^ in meters).^10^ Added to this, the CONUT score accounts for serum albumin, cholesterol, and total lymphocyte count to indicate measures of malnutrition.^11^

In order to assess the severity of HF, plasma brain natriuretic peptide (BNP) and its N‐terminal fragment [N‐terminal‐pro B‐type natriuretic peptide (NT‐proBNP)] have been routinely used as diagnostic biomarkers for cardiac dysfunction.^12^ Patients with HF are also characterized by increased C‐reactive protein (CRP) levels that are associated with higher congestion, worse prognosis, and increased mortality risk.^13^ The magnitude by which malnutrition may exacerbate these markers in this population has yet to be elucidated. In this systematic review and meta‐analysis, we therefore aimed to explore the association of malnutrition compared with normal nutrition in patients with HF, on plasma natriuretic peptide and CRP levels.

## Methods

The revised 2020 Preferred Reporting Items for Systematic Reviews and Meta‐Analyses (PRISMA) criteria were followed to conduct this systematic review and meta‐analysis.^14^ The protocol has been registered in the International Prospective Register of Systematic Reviews (PROSPERO) (CRD42023445076).

### Search strategy

From inception to July 2023, four databases (PubMed, Scopus, Web of Science and Cochrane Library) were independently searched by two investigators (K. P. and K. I.). The search phrases (‘Controlling Nutritional Status’ OR ‘CONUT’ OR ‘GNRI’ OR ‘geriatric nutritional risk index’ OR undernourished OR malnourished OR malnutrition OR undernutrition) AND (‘heart failure’ OR ‘ejection fraction’) were used (*Table* S1).

### Data extraction and risk of bias

Two investigators (K. P. and K. I.) extracted data independently, including the name of the first author, publication date, country of origin, participant age, study design, definition of malnutrition, sample size, participant sex and body mass index (BMI), left ventricular ejection fraction rate (LVEF), New York Heart Association classification and additional comorbidities. Disagreements were resolved by a third investigator (K. N.). The quality of the included studies was evaluated using the National Institute of Health (NIH) Quality Assessment Tool for Observational Cohort and Cross‐Sectional Studies, which consists of 14 questions regarding study quality rating (good, fair or poor).^15^

### Certainty of the evidence

The evaluation of evidence certainty was performed utilizing the Grading of Recommendations, Assessment, Development, and Evaluations (GRADE) framework.^16^ We investigated the following: the impact of malnutrition versus well‐nourished status on BNP, NT‐proBNP, and CRP levels, with malnutrition status determined by GNRI and CONUT scores. The certainty of evidence for mean differences in biomarker levels was graded across all comparisons.

### Statistical analysis

Quantitative data were considered as continuous measurements, and differences in outcomes between those with malnutrition versus normal nutrition were compared to determine mean differences (MDs) in levels of BNP, NT‐proBNP and CRP concentrations. When studies reported interquartile ranges (IQR), the formula ‘standard deviation (*SD*) = width of IQR/1.35’ was used to estimate missing *SD*s.^17^ The random‐effects model and the inverse‐variance approach were used to determine statistical significance.

Statistical heterogeneity of outcome measurements across studies was measured using the overlap of their confidence intervals (95) and expressed as Cochran's *Q* (*χ* ^2^ test) and *I* ^2^ measurements. Low heterogeneity was defined as *I* ^2^ between 30% and 49%, moderate heterogeneity between 50% and 74%, and high heterogeneity between 75% and above.^18^ In the case of substantial heterogeneity, a random‐effects meta‐regression was carried out to investigate potential sources of variability that could alter estimate rates across studies.^19^ Subgroup analyses based on overall malnutrition (scores ≥2) and mild malnutrition (scores 2–4) were conducted regarding CONUT scores, and ≤98 versus >98 scores in relation to GNRI. Furthermore, sensitivity analyses were performed to assess the robustness of reported statistical results by controlling for existing comorbidities, which differed between patients with and without malnutrition, as well as the risk of bias in the included studies. The meta‐analysis was synthesized using Review Manager (RevMan 5.4.1) software and a *P* value of <0.05 was considered statistically significant. Meta‐regressions were conducted using a random‐effects model to assess unexplained variance among studies with significant heterogeneity. The meta‐regression included factors such as age, BMI and LVEF. To assess the risk of publication bias in our analysis, we planned to generate funnel plots and visually inspect them for asymmetry. Additionally, we intended to quantitatively evaluate funnel plot asymmetry using Egger's weighted regression test. However, this quantitative assessment method was contingent upon having more than 10 studies in any comparison, a criterion that was not met in this study.

## Results

**Figure 1:**
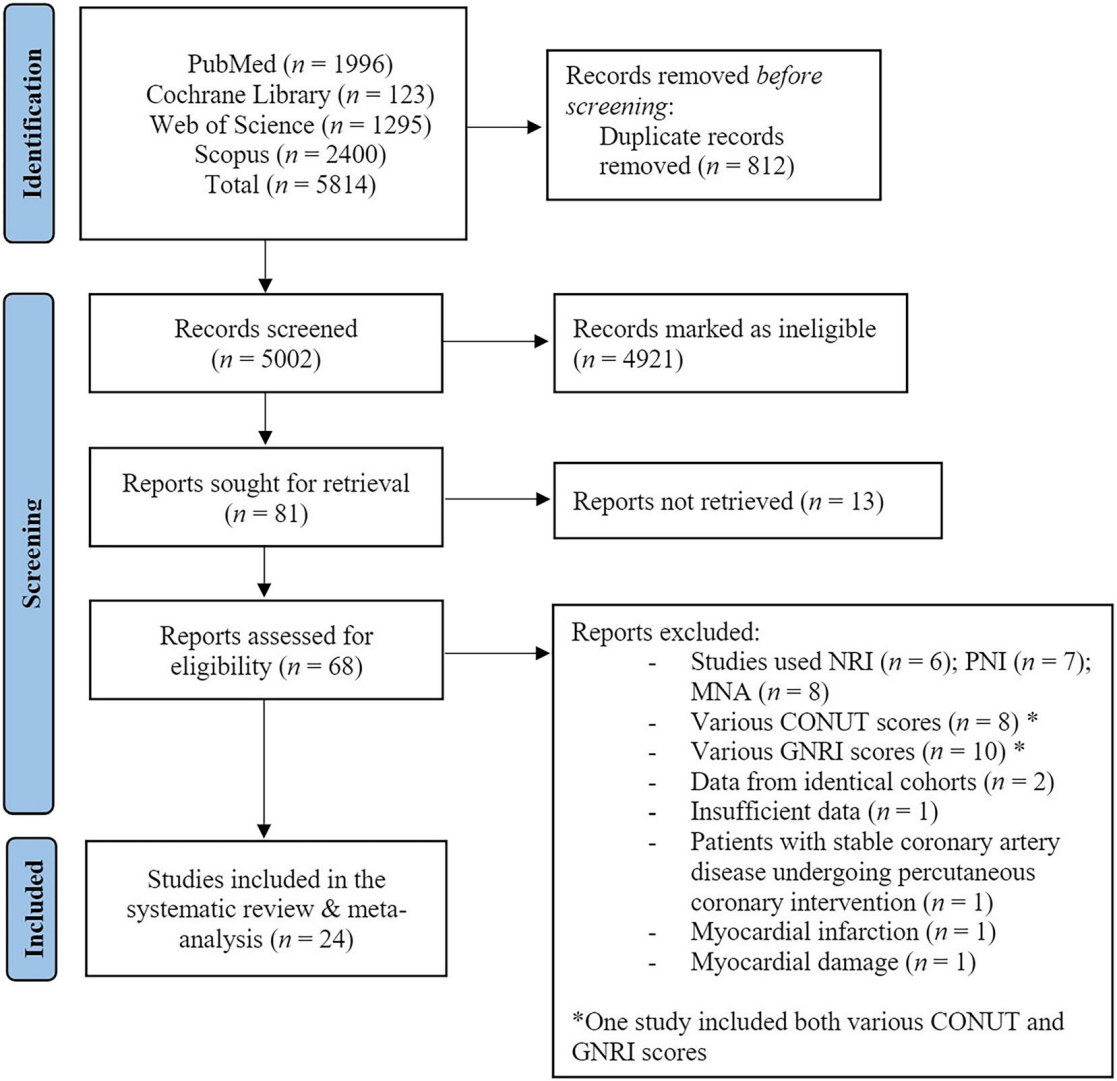
Literature search of the employed search terms. CONUT, Controlling Nutritional Status; GNRI, Geriatric Nutritional Risk Index; MNA, Mini Nutritional Assessment; PNI, Prognostic Nutritional Index.

The initial literature search provided 5814 publications. Following the exclusion of duplicates, abstracts, studies that full text could not be obtained and in a different language, 68 full texts were identified as eligible for inclusion in the systematic review and meta‐analysis. Of these 68 articles, 8 studies used the Mini Nutritional Assessment (MNA) score,^20^, ^21^, ^22^, ^23^, ^24^, ^25^, ^26^, ^27^ 7 used the Prognostic Nutritional Index (PNI) score^28^, ^29^, ^30^, ^31^, ^32^, ^33^, ^34^ and 6 used the Nutrition risk index (NRI) score.^35^, ^36^, ^37^, ^38^, ^39^, ^40^ In addition, 8 studies used various classifications of CONUT scores to identify multiple degrees of malnutrition,^41^, ^42^, ^43^, ^44^, ^45^, ^46^, ^47^, ^48^ and similarly, 10 studies used various GNRI scores.^43^, ^49^, ^50^, ^51^, ^52^, ^53^, ^54^, ^55^, ^56^, ^57^ The study by Horiuchi *et al*.^43^ included both various CONUT and GNRI scores. Furthermore, two studies were excluded due to identical cohorts as the ones included in our study,^58^, ^59^ one study had insufficient data,^60^ and studies that included patients that were not diagnosed with HF but had stable coronary artery disease undergoing percutaneous coronary intervention,^61^ myocardial infarction^62^ and myocardial damage.^63^ Overall, 24 studies were included in the systematic review and meta‐analysis (*Figure* *1* *)*.

### Descriptive results

Fifteen studies utilized the CONUT score from which seven explored the impact of a score 0–1 (normal nutrition) versus ≥2 (overall malnutrition),^64^, ^65^, ^66^, ^67^, ^68^, ^69^ while eight studies used scores of 0–1 (normal nutrition) versus 2–4 (mild malnutrition).^70^, ^71^, ^72^, ^73^, ^74^, ^75^, ^76^, ^77^ Twelve studies explored the relationship of malnutrition versus normal nutrition using the GNRI, for which eight studies^78^, ^79^, ^80^, ^81^, ^82^, ^83^, ^84^, ^85^ used a score <92 versus ≥92, while two studies^86^, ^87^ used scores of ≤98 versus >98. Higher scores pertinent to GNRI indicated better nutritional status. Detailed characteristics of the included studies are shown in *Table*s 1 and 2.

**Table 1:** Study and participant characteristics of the included studies assessing malnutrition through the CONUT score.

Study, year	Country	Study design		Malnutrition				Normal nutrition			
			Total *n*	*n* (M/F)	Age	LVEF%	NYHA III/IV	*n* (M/F)	Age	LVEF%	NYHA III/IV
Abulimiti *et al*. 2023	Japan	Prospective study	101	64 (46/18)	66 ± 25	36.6 ± 11.4	19	27 (20/7)	53 ± 19	32.5 ± 8.4	4
Chen *et al*. 2023	China	Prospective study	218	178 (121/57)	86 (81–90)	62 (57–64)	—	40 (18/22)	84 (78–90)	61 (58–64)	—
Zhao *et al*. 2023	China	Retrospective study	144	102 (63/39)	65.8 ± 17.4	31.5 ± 6.6	102	42 (36/6)	65.7 ± 13.2	33.9 ± 5.7	42
Jia *et al*. 2022	China	Retrospective study	380	237 (138/99)	66.0 ± 14.0	All: 36.1 ± 15.0	—	143 (77/66)	61.1 ± 14.5	All: 36.1 ± 15.0	—
Kinugasa *et al*. 2022	Japan	Prospective study	150	81 (54/27)	69.0 (63.0–78.0)	45.0 (27.5–56.5)	—	69 (50/19)	65.0 (55.0–73.0)	47.5 (34.5–57.1)	—
Uemura *et al*. 2022	Japan	Retrospective study	465	380 (219/161)	75.9 ± 12.9	42.0 ± 16.2	—	85 (49/36)	69.8 ± 13.9	42.0 ± 16.9	—
Ikeya *et al*. 2021	Japan	Retrospective study	190	132 (107/25)	69 (60–77)	30 (22–37)	112	58 (37/21)	67 (57–75)	29 (23–36)	45
Rubio‐Gracia *et al*. 2021	Spain	Retrospective study	102	89 (42/47)	80.6 (73.2–94.6)	50.5 (40.0–61.5)	18	13 (5/8)	79.6 (75.7–84.0)	63.5 (32.5–65.0)	2
Takada *et al*. 2021	Japan	Retrospective study	1,425	1,213 (1,099/214)	73 ± 14	41 ± 13	944	212 (140/72)	62 ± 15	38 ± 12	104
Shirakabe *et al*. 2018	Japan	Retrospective study	307	179 (115/64)	76 (68–83)	41 (29–51)	132	128 (87/41)	74 (64–79)	40 (29–51)	108
Alvarez‐Alvarez *et al*. 2018	Spain	Retrospective study	247	99 (79/20)	71 ± 9	39 ± 12	78	148 (109/39)	68 ± 10	41 ± 13	105
La Rovere *et al*. 2017	Italy	Retrospective study	466	251 (218/33)	63.3 ± 9.9	33.0 ± 10.4	169	215 (183/32)	59.0 ± 11.7	34.5 ± 10.7	107
Bermejo *et al*. 2017	Spain	Retrospective study	123	75 (49/26)	69.8 ± 11.1	49 had <50%	71	48 (27/21)	69.6 ± 11.0	35 had <50%	43
Nishi *et al*. 2017	Japan	Retrospective study	482	352 (219/133)	72.8 ± 13.3	42.5 (27.5–52.5)	312	130 (79/51)	68.8 ± 14.0	37.5 (27.5–47.5)	115

*Note*: Data are expressed as mean (standard deviation) or median (IQR).

Abbreviations: F, females; IQR, interquartile range; LVEF, left ventricular ejection fraction; M, males; NYHA, New York Heart Association.

**Table 2:** Study and participant characteristics of the included studies assessing malnutrition through GNRI.

Study, year	Country	Study design		Malnutrition				Normal nutrition			
			Total *n*	*n* (M/F)	Age	LVEF%	NYHA III/IV	*n* (M/F)	Age	LVEF%	NYHA III/IV
Hirose *et al*. 2021	Japan	Prospective study	890	414 (232/182)	82 (76–87)	47 ± 17	60	476 (289/187)	78 (72–84)	45 ± 16	49
Yasumura *et al*. 2020	Japan	Retrospective study	213	97 (50/47)	84 ± 7	49 ± 17	—	106(72/3 4)	79 ± 9	52 ± 18	—
Hirose *et al*. 2020	Japan	Retrospective study	181	96 (66/30)	71.0 ± 12.0	27.1 ± 7.1	85	105 (76/29)	63.6 ± 15.3	27.0 ± 6.8	90
Nishi *et al*. 2019	Japan	Retrospective study	110	49 (29/20)	80.4 ± 7.7	58.3 (53.2–64.7)	44	61 (30/31)	77.0 ± 6.5	61.6 (55.9–68.7)	56
Kawakubo *et al*. 2022	Japan	Retrospective study	1,231	525 (325/200)	78 (69–84)	30 (25–35)	432	706 (547/159)	68 (57–77)	30 (23–35)	553
Kitamura *et al*. 2019	Japan	Retrospective study	96	38 (17/21)	83.5 ± 8.3	49.7 ± 15.0	30	58 (32/26)	81.0 ± 6.6	45.4 ± 15.8	46
Sze *et al*. 2019	United Kingdom	Prospective study	952	132 (83/49)	80 (74–84)	46% < 40 LVEF%	60	820 (572/248)	74 (66–80)	38% < 40 LVEF%	257
Sargento *et al*. 2017	Portugal	Retrospective study	143	16 (9/7)	78.4 ± 7.6	30 ± 7.9	9	127(36/9 1)	75 ± 6.3	28.7 ± 7.8	42
Honda *et al*. 2016	Japan	Retrospective study	490	162 (94/68)	81 ± 8	39 (24–54)	129	328 (193/135)	78 ± 7	40 (25–55)	273
Kinugasa *et al*. 2013	Japan	Retrospective study	152	73 (33/40)	78 ± 11	57.1 ± 11.6	64	79 (45/34)	76 ± 10	56.4 ± 10.6	63

*Note*: Data are expressed as mean (standard deviation) or median (IQR).

Abbreviations: F, females; IQR, interquartile range; LVEF, left ventricular ejection fraction; M, males; NYHA, New York Heart Association.

### BNP levels in patients with HF and malnutrition versus normal nutrition

**Figure 2:**
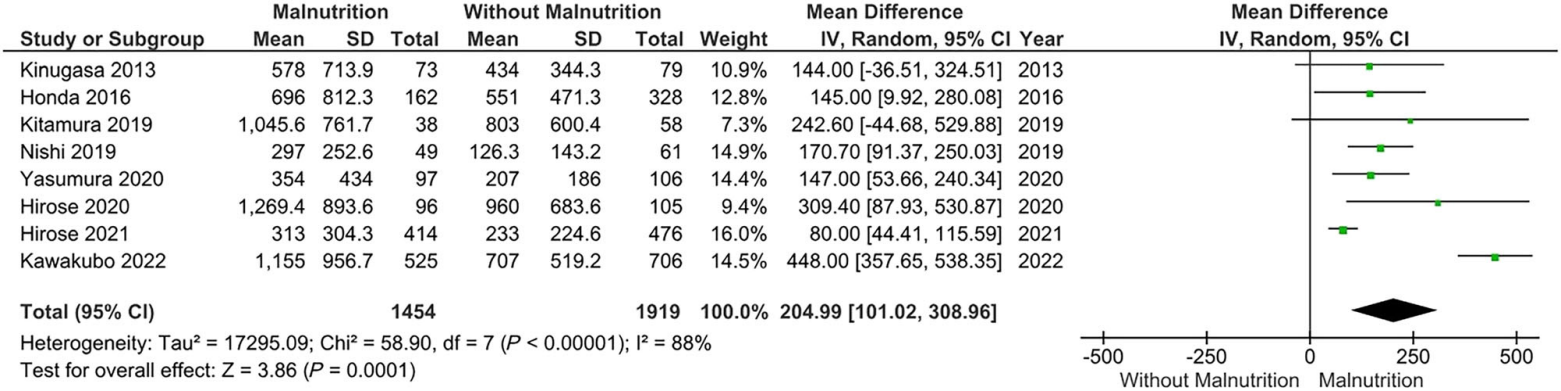
Association of brain natriuretic peptide levels in relation to malnutrition versus normal nutrition in heart failure, using the Geriatric Nutritional Risk Index scores. CI, confidence interval; *SD*, standard deviation.

Our main analysis utilizing the GNRI (*k* = 8; *n* = 1454 with malnutrition and *n* = 1919 without malnutrition) showed that malnutrition was associated with significantly higher levels of BNP [MD: 204.99, 95% CI (101.02–308.96, *I* ^2^ = 88%, *P* < 0.01) (*Figure* 2)]. Our sensitivity analysis, excluding those with malnutrition and more comorbidities (valvular disease), did not alter the results [MD: 215.48, 95% CI (92.19–338.76, *I* ^2^ = 90%, *P* < 0.01) (*Figure* S1)] nor when we excluded those with malnutrition and higher prevalence of valvular disease and prior admission for HF [MD: 228.48, 95% CI (85.52–371.45, *I* ^2^ = 92%, *P* < 0.01) (Figure S2)]. Exclusion of two studies due to increased risk of bias did not alter the findings of the main analysis [MD: 240.74, 95% CI (104.74–376.75, *I* ^2^ = 81%, *P* < 0.01) (Figure S3)].

**Figure 3:**
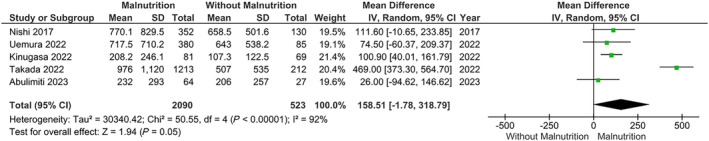
Association of brain natriuretic peptide levels in relation to malnutrition versus normal nutrition in heart failure, using the Controlling Nutritional Status scores. CI, confidence interval; *SD*, standard deviation.

Our main analysis using the CONUT score (0–1 vs. ≥2) (*k* = 5; *n* = 2090 with overall malnutrition and *n* = 523 without malnutrition) showed statistically insignificantly greater levels of BNP during overall malnutrition [MD: 158.51, 95% CI (−1.78–318.79, *I* ^2^ = 92%, *P* = 0.05) (*Figure* 3)]. Sensitivity analysis excluding a study for which patients with malnutrition had higher prevalence of acute infection, malignancy, and frailty also displayed insignificant associations [MD: 178.11, 95% CI (−14.47–370.68, *I* ^2^ = 94%, *P* = 0.07) (Figure S4)]. When we compared mild malnutrition (CONUT scores of 2–4) versus normal nutrition no associations were depicted [*k* = 3; MD: 95.24, 95% CI (−37.24–227.72, *I* ^2^ = 54%, *P* = 0.16) (Figure S5)] while similar findings were also observed by excluding a study with increased risk of bias [MD: 190.61, 95% CI (0.82–380.41, *I* ^2^ = 93%, *P* = 0.05) (Figure S6)].

### NT‐proBNP levels in patients with HF and malnutrition versus normal nutrition

**Figure 4:**
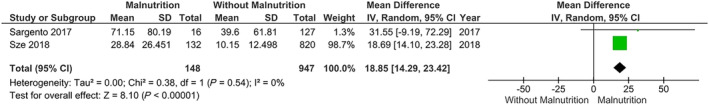
Association of N‐terminal‐pro B‐type natriuretic peptide levels in relation to malnutrition versus normal nutrition in heart failure, using the Geriatric Nutritional Risk Index scores. CI, confidence interval; *SD*, standard deviation.

**Figure 5:**
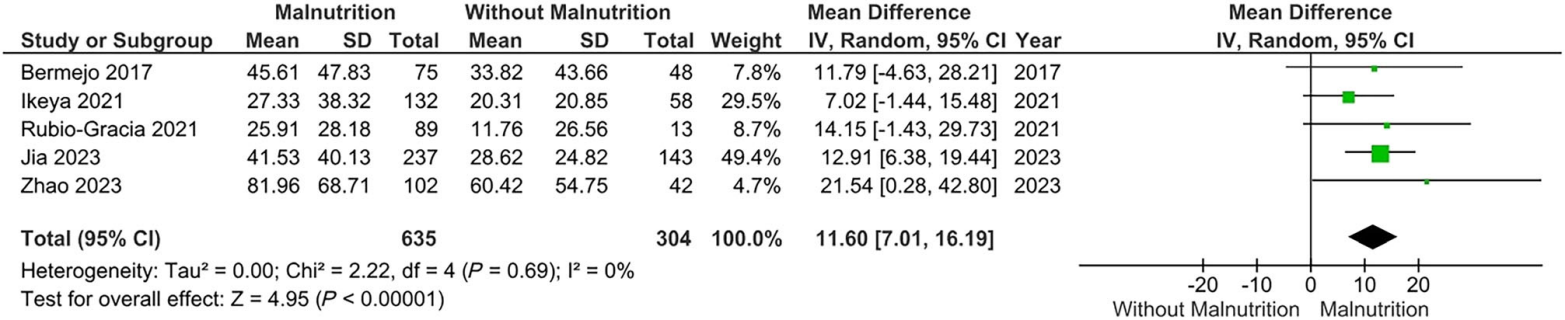
Association of N‐terminal‐pro B‐type natriuretic peptide levels in relation to malnutrition versus normal nutrition in heart failure, using the Controlling Nutritional Status scores. CI, confidence interval; *SD*, standard deviation.

Our main analysis using the GNRI [*k* = 2; *n* = 148 with malnutrition (score >98) and *n* = 947 without malnutrition (score ≤98)] showed that malnutrition was associated with significantly higher levels of NT‐proBNP [MD: 1885.14, 95% CI (1428.76–2341.52, *I* ^2^ = 0%, *P* < 0.01) (*Figure* *4*; divided by 100 as RevMan could not show values above 1000)]. Through CONUT, mild malnutrition (*n* = 635) also exhibited significantly higher levels of NT‐proBNP compared with normal nutrition (*n* = 304) [MD: 1160.05, 95% CI (701.04–1619.07, *I* ^2^ = 0%, *P* < 0.01) (*Figure* *5*; divided by 100)]. Identical results were shown after the omission of two studies with increased risk of bias [MD: 1004.84, 95% CI (303.15–1706.53, *I* ^2^ = 0%, *P* < 0.01) (Figure S7)].

### CRP levels in patients with HF and malnutrition versus normal nutrition

**Figure 6:**
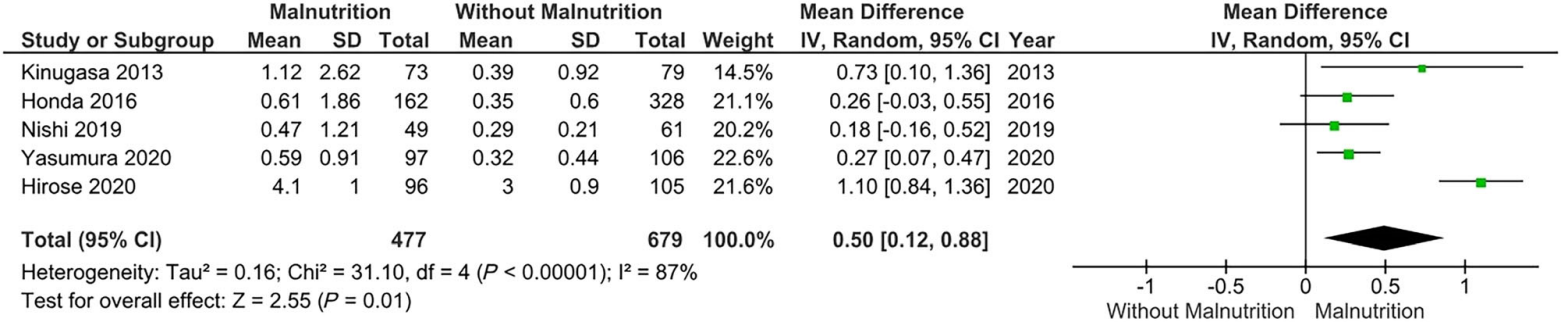
Association of C‐reactive protein levels in relation to malnutrition versus normal nutrition in heart failure, using the Geriatric Nutritional Risk Index scores. CI, confidence interval; *SD*, standard deviation.

The main analysis based on GNRI showed that patients with normal nutrition (*n* = 679) had significantly lower levels of CRP compared with those with malnutrition (*n* = 477) [*k* = 5; MD: 0.50, 95% CI (0.12–0.88, *I* ^2^ = 87%, *P* = 0.01) (*Figure* 6)]. Considering the multiple studies for which those with malnutrition had a higher number of comorbidities, several sensitivity analyses were conducted. Sensitivity analyses excluding those with higher rate of valvular disease [MD: 0.56, 95% CI (0.06–1.06, *I* ^2^ = 88%, *P* = 0.03) (Figure S8)], previous hospitalization due to HF [MD: 0.56, 95% CI (0.08–1.04, *I* ^2^ = 90%, *P* = 0.02) (Figure S9)], valvular disease and previous hospitalization due to HF [MD: 0.67, 95% CI (0.02–1.32, *I* ^2^ = 88%, *P* = 0.04) (Figure S10)], haemodialysis [MD: 0.50, 95% CI (0.12–0.88, *I* ^2^ = 87%, *P* = 0.01) (Figure S11)], haemodialysis, valvular disease and previous hospitalization due to HF [MD: 0.67, 95% CI (0.02–1.32, *I* ^2^ = 88%, *P* = 0.04) (Figure S12)] showed identical outcomes to our original analysis. Similar findings were depicted following exclusion of one study with increased risk of bias [MD: 0.58, 95% CI (0.12–1.03, *I* ^2^ = 89%, *P* = 0.01) (Figure S13)].

**Figure 7:**
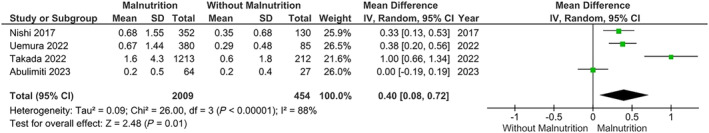
Association of C‐reactive protein levels in relation to malnutrition versus normal nutrition in heart failure, using the Controlling Nutritional Status scores. CI, confidence interval; *SD*, standard deviation.

Our main analysis according to CONUT scores showed that those will overall malnutrition (*n* = 2009) had significantly higher levels of CRP versus those with normal nutritional status (*n* = 454) (*k* = 4; MD: 0.40, 95% CI 0.08–0.72, *I* ^2^ = 88%, *P* = 0.01) (*Figure* *7*). Sensitivity analysis excluding a study for which patients with overall malnutrition had higher prevalence of acute infection, malignancy and frailty, no statistically significant differences between groups were observed [MD: 0.42, 95% CI (−0.06–0.90, *I* ^2^ = 92%, *P* = 0.08) (Figure S14)]. Exclusion of one study with increased risk of bias did not alter the findings of the main analysis [MD: 0.54, 95% CI (0.21–0.86, *I* ^2^ = 83%, *P* < 0.01) (Figure S15)].

### Risk of bias of included studies

Regarding CONUT scores, six of the included studies were considered poor^64^, ^70^, ^72^, ^74^, ^75^, ^77^ in relation to risk of bias risk, four studies were marked as fair,^66^, ^67^, ^68^, ^71^ and four studies as good^65^, ^69^, ^73^, ^76^ (Table S2). Regarding GNRI, two studies were evaluated as poor,^78^, ^79^ four studies as fair,^81^, ^82^, ^83^, ^86^ and four studies as good^80^, ^84^, ^85^, ^87^ (Table S3).

### Certainty of the evidence

The evaluation of evidence certainty is detailed in the Table S4 and S5, with assessments made distinctively for malnutrition as determined by GNRI and CONUT scores. For comparisons utilizing GNRI scores, evidence certainty varied from low to moderate. In contrast, comparisons using CONUT scores exhibited certainty levels ranging from very low to low. The evidence certainty for the association between CONUT scores and BNP levels was downgraded owing to imprecision. Conversely, for differences in NT‐proBNP levels assessed by GNRI score, the certainty was elevated due to the observed large effect size.

### Meta‐regression analyses

The high heterogeneity observed for the differences in BNP and CRP following the assessment of GNRI and CONUT scores for those with (mild) malnutrition versus normal nutrition was further explored by employing multiple meta‐regression analyses. Differences in age, LVEF and BMI did not explain the potentially increased heterogeneity among studies for plasma levels of BNP and CRP in relation to CONUT. Similar results were shown regarding GNRI scores; however, age was shown to be a significant covariate that could affect changes in CRP [*r* = −0.1238, *SE* = 0.0616, 95% CI (−0.24 to −0.00, *z* = −2.01, *P* = 0.04)]. Full details are available in Table S6.

## Discussion

In this systematic review and meta‐analysis, normal nutrition scores based on CONUT and GNRI in patients with HF were associated with lower levels of BNP, NT‐proBNP and CRP levels compared with patients with malnutrition. Age was shown to mediate CRP levels following meta‐regression in relation to GNRI.

In patients with HF, multiple factors, including age, BMI, renal function, gender and comorbidities such as atrial fibrillation, may influence BNP and NT‐proBNP levels.^88^, ^89^ Notably, individuals with obesity often exhibit decreased BNP/NT‐proBNP levels, possibly due to increased neprilysin pathway activity, which is critical for BNP clearance and glomerular hyperfiltration.^90^ Conversely, patients with cachexia that are accompanied by increased prevalence of malnutrition, typically show elevated BNP/NT‐proBNP levels, with HF‐induced gut oedema and hypoalbuminemia potentially worsening malnutrition through nutrient malabsorption.^91^ While the link between malnutrition and HF is apparent, its specific impact on BNP/NT‐proBNP levels remains elusive.

Therefore, although we observed a substantial elevation in BNP and NT‐proBNP levels in two malnutrition scores with minor heterogeneity, these findings suggest the need to consider the modifying effect of malnutrition when interpreting BNP/NT‐proBNP values.

Moreover, HF disrupts the balance between anabolic and catabolic metabolism through inflammatory pathways.^91^ In conjunction with malnutrition, inflammation has been identified as a prognostic factor in this population.^41^ Although CRP serves as a surrogate marker of inflammation, its value may be influenced by factors such as age, sex, infections and comorbidities.^92^ In our study, CRP levels were markedly elevated in patients with HF and malnutrition compared with their non‐malnourished counterparts, demonstrating slightly more pronounced values in the GNRI score. Additionally, our findings suggest that age may act as a mediator between CRP and malnutrition assessed via GNRI, implying that GNRI, originally designed for older patients,^93^ may be more responsive to higher inflammation levels associated with ageing. In particular, although CONUT may be calculated solely based on serum markers, GNRI incorporates physical factors (height/weight), which are strongly influenced by ageing. Conclusively, our data suggest a prominent link between CRP and malnutrition, however, incorporation of assessment tools such as the GNRI score may alter this relationship, specifically in older patients.

### Strengths and limitations

This is the first meta‐analysis investigating the link between malnutrition with natriuretic peptides and a marker of inflammation. Moreover, the exploration of two distinct malnutrition scoring systems unveiled factors demanding specific attention in malnutrition assessment within this clinical cohort, potentially impeding the precise correlation with inflammatory profiles. This study was also prone to several limitations. Initially, the differentiation between HF with reduced and preserved ejection fraction, characterized by disparate natriuretic peptide levels, was unattainable due to data unavailability. Likewise, the potential impact of inpatient versus outpatient status, with potential variations in settings, rehabilitation protocols and HF severity, could not be established for the same reason. Additionally, our analyses were grounded in cross‐sectional data; therefore, causal (bidirectional) relationships may not be established. The generalizability of our findings is limited as most of the included studies enrolled Asian patients. This limitation is highlighted by Takeuchi *et al*.,^53^ who found that Japanese HF patients eligible for the EMPEROR‐Preserved trial had lower BMI than the primarily Western group in the actual trial, underscoring differences in malnutrition assessment using GNRI between Asian and Western populations. This suggests the need for cautious application of our results across different ethnicities.

## Conclusions

Our systematic review and meta‐analysis reveals that normal nutrition scores in patients with HF are linked to lower BNP, NT‐proBNP, and CRP levels compared with malnourished counterparts. Integrating BNP/NT‐proBNP and CRP monitoring in conjunction with nutritional status assessment may provide insights related to HF trajectory, aiding in more supportive patient management. Enhanced nutritional support, particularly targeted towards malnourished patients with HF, may emerge as a clinically valuable strategy, improving overall care.

## Conflict of interest

None declared.

## Supporting information


**Data S1.** Supporting Information.

**Table S1.** Search terms employed in the screening based on title, abstract, and keywords in the literature search.
**Table S2.** Risk of bias assessment using CONUT scores.
**Table S3.** Risk of bias assessment using GNRI scores.
**Table S4.** Summary of findings for comparisons using GNRI score.
**Table S5.** Summary of findings for comparisons using CONUT score.
**Table S6.** Meta‐regression analyses to explore the impact of potential covariates.
**Figure S1.** Mean differences in BNP levels according to malnutrition status established with the use of GNRI in heart failure patients after exclusion of studies with higher rate of comorbid valvular disease in malnourished patients. Mean differences are presented as 95% confidence intervals using random effects model.
**Figure S2.** Mean differences in BNP levels according to malnutrition status established with the use of GNRI in heart failure patients after exclusion of studies with higher prevalence of valvular disease and prior HF admission in the malnourished group. Mean differences are presented as 95% confidence intervals using random effects model.
**Figure S3.** Mean differences in BNP levels according to malnutrition status established with the use of GNRI in heart failure patients after exclusion of studies with high risk of bias. Mean differences are presented as 95% confidence intervals using random effects model.
**Figure S4.** Mean differences in BNP levels according to malnutrition status established with the use of CONUT score in heart failure patients after exclusion of studies with higher prevalence of acute infection, malignancy, and frailty in the malnourished group. Mean differences are presented as 95% confidence intervals using random effects model.
**Figure S5.** Mean differences in BNP levels in patients with mild malnutrition (CONUT scores of 2–4) compared to normal nutrition and heart failure. Mean differences are presented as 95% confidence intervals using random effects model.
**Figure S6.** Mean differences in BNP levels according to malnutrition status established with the use of CONUT score in heart failure patients after exclusion of studies with high risk of bias. Mean differences are presented as 95% confidence intervals using random effects model.
**Figure S7.** Mean differences in NT‐proBNP levels according to malnutrition status established with the use of CONUT in heart failure patients after exclusion of studies with high risk of bias. Mean differences are presented as 95% confidence intervals using random effects model.
**Figure S8.** Mean differences in CRP levels according to malnutrition status established with the use of GNRI in heart failure patients after exclusion of studies with higher prevalence of valvular disease in the malnourished group. Mean differences are presented as 95% confidence intervals using random effects model.
**Figure S9.** Mean differences in CRP levels according to malnutrition status established with the use of GNRI in heart failure patients after exclusion of studies with higher prevalence of prior HF admission in the malnourished group. Mean differences are presented as 95% confidence intervals using random effects model.
**Figure S10.** Mean differences in CRP levels according to malnutrition status established with the use of GNRI in heart failure patients after exclusion of studies with higher prevalence of valvular disease and prior HF admission in the malnourished group. Mean differences are presented as 95% confidence intervals using random effects model.
**Figure S11.** Mean differences in CRP levels according to malnutrition status established with the use of GNRI in heart failure patients after exclusion of studies with higher prevalence of haemodialysis in the malnourished group. Mean differences are presented as 95% confidence intervals using random effects model.
**Figure S12.** Mean differences in CRP levels according to malnutrition status established with the use of GNRI in heart failure patients after exclusion of studies with higher prevalence of haemodialysis, valvular disease, and previous hospitalization due to HF in the malnourished group. Mean differences are presented as 95% confidence intervals using random effects model.
**Figure S13.** Mean differences in CRP levels according to malnutrition status established with the use of GNRI in heart failure patients after exclusion of one study with high risk of bias. Mean differences are presented as 95% confidence intervals using random effects model.
**Figure S14.** Mean differences in CRP levels according to malnutrition status established with the use of CONUT scores in heart failure patients after exclusion of studies with higher prevalence of acute infection, malignancy, and frailty in the malnourished group. Mean differences are presented as 95% confidence intervals using random effects model.
**Figure S15.** Mean differences in CRP levels according to malnutrition status established with the use of CONUT score in heart failure patients after exclusion of one study with high risk of bias. Mean differences are presented as 95% confidence intervals using random effects model.

## Data Availability

Data are available upon request.
