# Correction to ‘Natriuretic peptides and C‐reactive protein in in heart failure and malnutrition: A systematic review and meta‐analysis’

**DOI:** 10.1002/ehf2.15210

**Published:** 2025-01-14

Prokopidis, K. , Irlik, K. , Ishiguchi, H. , Rietsema, W. , Lip, G. Y. H. , Sankaranarayanan, R. , Isanejad, M. , and Nabrdalik, K. (2024) Natriuretic peptides and C‐reactive protein in in heart failure and malnutrition: a systematic review and meta‐analysis. ESC Heart Failure, 11: 3052–3064. 10.1002/ehf2.14851 38850122 PMC11424355

The article title contains a typo in the published version.

The correct article title is as follows:

Natriuretic peptides and C‐reactive protein in heart failure and malnutrition: A systematic review and meta‐analysis

We apologize for this error.

