# Supplementary material for: Natriuretic peptides and C‐reactive protein in in heart failure and malnutrition: a systematic review and meta‐analysis
Source: ESC Heart Fail. 2024 Jun 8;11(5):3052–64. doi: 10.1002/ehf2.14851 (PMC11424355; doi:10.1002/ehf2.14851)

**Supplementary material**

Table of Contents

**Search strategy1**

**Risk of Bias Assessment3**

Studies using CONUT score3

Studies using GNRI score33

**Certainty of the evidence55**

**Meta-regression analyses57**

**Sensitivity analyses58**

**Search strategy**

**Table S1.**Search terms employed in the screening based on title, abstract, and keywords in the literature search.

| **Database** | **Search terms** |
| --- | --- |
|  |  |
| PubMed | ("Controlling Nutritional Status" OR "CONUT" OR "GNRI" OR "geriatric nutritional risk index" OR undernourished  OR malnourished OR malnutrition OR undernutrition) AND (“heart failure” OR “ejection fraction”) |
| Cochrane Library | ("Controlling Nutritional Status" OR "CONUT" OR "GNRI" OR "geriatric nutritional risk index" OR undernourished  OR malnourished OR malnutrition OR undernutrition) AND (“heart failure” OR “ejection fraction”) |
| Web of Science | ("Controlling Nutritional Status" OR "CONUT" OR "GNRI" OR "geriatric nutritional risk index" OR undernourished  OR malnourished OR malnutrition OR undernutrition) AND (“heart failure” OR “ejection fraction”) |
| Scopus | TITLE-ABS-KEY (("Controlling Nutritional Status" OR "CONUT" OR "GNRI" OR "geriatric nutritional risk index"  OR undernourished OR malnourished OR malnutrition OR undernutrition) AND ("heart failure" OR "ejection fraction")) |

| Study  Year | Q1 | Q2 | Q3 | Q4 | Q5 | Q6 | Q7 | Q8 | Q9 | Q10 | Q11 | Q12 | Q13 | Q14 | Overall |
| --- | --- | --- | --- | --- | --- | --- | --- | --- | --- | --- | --- | --- | --- | --- | --- |
| Abulimiti 2023 | Y | Y | Y | Y | N | Y | Y | N | CD | N | N | CD | N | N | Poor |
| Alvarez-Alvarez 2018 | Y | Y | Y | Y | N | Y | N | Y | N | Y | N | NR | NR | Y | Poor |
| Bermejo 2017 | Y | Y | NR | Y | N | Y | N | Y | NR | N | N | NR | NR | N | Poor |
| Chen 2023 | Y | Y | Y | Y | N | Y | Y | Y | N | N | Y | NR | NR | Y | Poor |
| Ikeya 2021 | Y | Y | Y | Y | N | Y | Y | Y | Y | N | N | Y | NR | Y | Good |
| Jia 2023 | N | N | NA | N | N | Y | N | Y | Y | N | N | NR | NR | CD | Poor |
| Kinugasa 2022 | Y | Y | NR | Y | N | Y | Y | N | Y | N | N | NR | NR | Y | Fair |
| La Rovere 2017 | Y | Y | Y | Y | N | Y | Y | N | Y | N | Y | NR | Y | N | Good |
| Nishi 2017 | Y | Y | Y | Y | N | Y | Y | Y | Y | N | NR | NR | NR | N | Fair |
| Rubio-Garcia 2021 | Y | Y | NR | Y | N | Y | Y | Y | Y | N | CD | NR | CD | Y | Fair |
| Shirakabe 2018 | N | Y | N | Y | N | Y | Y | Y | Y | N | Y | NR | NR | N | Poor |
| Takada 2021 | Y | Y | Y | Y | N | Y | Y | N | Y | N | Y | NR | Y | N | Fair |
| Uemura 2022 | Y | Y | Y | Y | N | Y | Y | Y | Y | N | Y | NR | N | Y | Good |
| Zhao 2023 | Y | Y | Y | Y | N | Y | N | Y | Y | N | Y | NR | Y | Y | Good |

**Risk of bias assessment**

**Table S2.** Risk of bias assessment using CONUT scores.

## 01. Abulimiti 2023 [Juntendo University Hospital, Tokyo]

|  | Yes | No | Other (CD, NR, NA) |
| --- | --- | --- | --- |
| 1. Was the research question or objective in this paper clearly stated? | Y |  |  |
| 2. Was the study population clearly specified and defined?  🡺 see original study [Ishiwata 2022] LV systolic dysfunction hospitalised due to ADHF May 2012-Apr 2018. | Y |  |  |
| 3. Was the participation rate of eligible persons at least 50%?  Fig 1: 79/241 patients excluded re. missing data == 32.8% | Y |  |  |
| 4. Were all the subjects selected or recruited from the same or similar populations (including the same time period)? Were inclusion and exclusion criteria for being in the study prespecified and applied uniformly to all participants?  🡺 see Ishiwata | Y |  |  |
| 5. Was a sample size justification, power description, or variance and effect estimates provided?  🡺 neither in paper nor in Ishiwata |  | N |  |
| 6. For the analyses in this paper, were the exposure(s) of interest measured prior to the outcome(s) being measured?  🡺 Ishiwata – under Subjects: “After the initial improvement of AHF [acute HF], overnight polysomnography was performed”; under Other Data Collection: “Baseline data were collected prospectively at the time of the sleep study”. | Y |  |  |
| 7. Was the **timeframe** sufficient so that one could reasonably expect to see an association between exposure and outcome if it existed?  🡺 Ishiwata: recruitment May 2012 – April 2018; Abulimiti f/u till April 2019 🡺 for those recruited in the last year of the study, timeframe is short. | Y |  |  |
| 8. For exposures that can vary in amount or level, did the study examine different levels of the exposure as related to the outcome (e.g., categories of exposure, or exposure measured as continuous variable)?  🡺 It was malnutrition [>2] and none [1-2] .. malnutrition not graded |  | N |  |
| 9. Were the exposure measures (independent variables) clearly defined, valid, reliable, and implemented consistently across all study participants?  🡺 Baseline measurements for CONUT, lab methods not mentioned. Method of f/u |  |  | CD |
| 10. Was the exposure(s) assessed more than once over time? |  | N |  |
| 11. Were the outcome measures (dependent variables) clearly defined, valid, reliable, and implemented consistently across all study participants?  🡺 “Outcome measures were obtained by reviewing electronic medical records of our hospital.” In many hospitals [UK, NL], this would not be reliable, as hospitals don’t always get notified of the death of a patient. |  | N |  |
| 12. Were the outcome assessors blinded to the exposure status of participants?  🡺 not mentioned who reviewed hospital records and whether they knew of patients’ exposure. |  |  | CD |
| 13. Was loss to follow-up after baseline 20% or less?  🡺 Methods, Subjects: 162/241 pts analysed [rest had missing data] == 33%  Search loss/ lost = nil. |  | N |  |
| 14. Were key potential confounding variables measured and adjusted statistically for their impact on the relationship between exposure(s) and outcome(s)?  🡺 They say statins are confounding factor for cholesterol, haemodilution confounding for albumin. Statins not adjusted for. “After accounting for age, creatinine and BNP levels” [in statistics paragraph]  Statin use NS between groups – though in pts with Cheyne Stokes, statin use is 26 v. 51%] |  | N |  |

*CD, cannot determine; NA, not applicable; NR, not reported

| Quality rating (Good/ Fair/ Poor) | Poor |
| --- | --- |
| Rater 1 initials: | Fair |
| Rater 2 initials: | Poor |
| Additional comments, if Poor – why? | - Lost to f/u > 20% [33% due to missing data, no comment on lost to f/u after that] .. only that ‘all patients were followed up at Juntendo hospital every 1-2 months]. - Several questions CD - No adjusting for statin use. Email sent 10/7/2023 to [kasai-t@mx6.nisiq.net](mailto:kasai-t@mx6.nisiq.net).– no reply. |

## 02. Alvarez-Alvarez 2018 [Santiago de Compostela, Spain]

|  | Yes | No | Other (CD, NR, NA) |
| --- | --- | --- | --- |
| 1. Was the research question or objective in this paper clearly stated? | Y |  |  |
| 2. Was the study population clearly specified and defined? | Y |  |  |
| 3. Was the participation rate of eligible persons at least 50%?  🡺 Results, baseline char: 328 CRT devices implanted 26 not all data available -> 302 included. It seemed none met exclusion criteria, all of CRT patients otherwise met inclusion crit [which may have been the same as criteria for CRT implant] | Y |  |  |
| 4. Were all the subjects selected or recruited from the same or similar populations (including the same time period)? Were inclusion and exclusion criteria for being in the study prespecified and applied uniformly to all participants? | Y |  |  |
| 5. Was a sample size justification, power description, or variance and effect estimates provided?  🡺 Limitations – sample size relatively small .. convenience sample of single centre. |  | N |  |
| 6. For the analyses in this paper, were the exposure(s) of interest measured prior to the outcome(s) being measured? | Y |  |  |
| 7. Was the timeframe sufficient so that one could reasonably expect to see an association between exposure and outcome if it existed?  🡺 Materials and methods: recruitment = Aug 2005 – Apr 2015. F/u at least 1 year. |  | N |  |
| 8. For exposures that can vary in amount or level, did the study examine different levels of the exposure as related to the outcome (e.g., categories of exposure, or exposure measured as continuous variable)? | Y |  |  |
| 9. Were the exposure measures (independent variables) clearly defined, valid, reliable, and implemented consistently across all study participants?  🡺 No lab methods are mentioned? were methods changed over time, e.g. new lab platform/ method? |  | N |  |
| 10. Was the exposure(s) assessed more than once over time? | Y |  |  |
| 11. Were the outcome measures (dependent variables) clearly defined, valid, reliable, and implemented consistently across all study participants?  🡺 No mention of loss to f/u, or even how mortality was assessed. |  | N |  |
| 12. Were the outcome assessors blinded to the exposure status of participants? |  |  | NR |
| 13. Was loss to follow-up after baseline 20% or less?  🡺 I have written to author [belenalvarez85@hotmail.com](mailto:belenalvarez85@hotmail.com) 11/7/23 – no answer as of 14/8/23 |  |  | NR |
| 14. Were key potential confounding variables measured and adjusted statistically for their impact on the relationship between exposure(s) and outcome(s)?  🡺 Multivariate analysis conducted [3.3] – age, male sex, low sodium, LA diameter were independent predictors of HF admission/death. Statins not even mentioned in the paper. | Y |  |  |

*CD, cannot determine; NA, not applicable; NR, not reported

| Quality rating (Good/ Fair/ Poor) | Poor |
| --- | --- |
| Rater 1 initials: | Poor |
| Rater 2 initials: | Poor |
| Additional comments, if Poor – why? | Several issues not reported, inc. blinding. there is a statement re. exclusion for missing data [26/328] but none about loss to f/u, how mortality was assessed. Statins don’t even get a mention in the paper. They might have better f/u data because patients had a device and would need to come back to lab to get it checked, but they are not saying it. |

## 03. Bermejo 2017 [Santiago de Compostela, Spain]

|  | Yes | No | Other (CD, NR, NA) |
| --- | --- | --- | --- |
| 1. Was the research question or objective in this paper clearly stated? | Y |  |  |
| 2. Was the study population clearly specified and defined? | Y |  |  |
| 3. Was the participation rate of eligible persons at least 50%?  Methods, 1^st^ para: exclusion if unknown nutritional status, but not how many. |  |  | NR |
| 4. Were all the subjects selected or recruited from the same or similar populations (including the same time period)? Were inclusion and exclusion criteria for being in the study prespecified and applied uniformly to all participants? | Y |  |  |
| 5. Was a sample size justification, power description, or variance and effect estimates provided? |  | N |  |
| 6. For the analyses in this paper, were the exposure(s) of interest measured prior to the outcome(s) being measured? | Y |  |  |
| 7. Was the timeframe sufficient so that one could reasonably expect to see an association between exposure and outcome if it existed?  🡺 Mean f/u 326 days – less than a year |  | N |  |
| 8. For exposures that can vary in amount or level, did the study examine different levels of the exposure as related to the outcome (e.g., categories of exposure, or exposure measured as continuous variable)? | Y |  |  |
| 9. Were the exposure measures (independent variables) clearly defined, valid, reliable, and implemented consistently across all study participants?  🡺 Not recorded exactly: [1] it says ‘ECG/ echo were *also* recorded < 24 hours of admission – does ‘also’ refer to bloods? .. so probably yes, but not exactly sure. Also, lab methods are not reported. One assumes though that bloods were done on day of admission. |  |  | NR |
| 10. Was the exposure(s) assessed more than once over time? |  | N |  |
| 11. Were the outcome measures (dependent variables) clearly defined, valid, reliable, and implemented consistently across all study participants?  🡺 2.2 “F/u methods involved one of the following: use of hospital records, hospital visits or general physician visits... no statement about loss to f/u. |  | N |  |
| 12. Were the outcome assessors blinded to the exposure status of participants? |  |  | NR |
| 13. Was loss to follow-up after baseline 20% or less?  No statement re. loss to f/u [search loss/ lost] |  |  | NR |
| 14. Were key potential confounding variables measured and adjusted statistically for their impact on the relationship between exposure(s) and outcome(s)?  🡺 statins not checked as confounders .. while 27% of patients had ischaemic HF. BBlockers, ACE and spironolactone were checked for confounding. |  | N |  |

*CD, cannot determine; NA, not applicable; NR, not reported

| Quality rating (Good/ Fair/ Poor) | Poor |
| --- | --- |
| Rater 1 initials: | Fair |
| Rater 2 initials: | Poor |
| Additional comments, if Poor – why? | Not reporting how many excluded for unknown nutritional status, and not reporting on loss to f/u, not adjusting for statin use. |

Mildly malnourished were more likely to have adverse outcome than severely malnourished.

## 04. Chen 2023 [Beijing] – HF with preserved EF in older adult coronary artery disease patients.

|  | Yes | No | Other (CD, NR, NA) |
| --- | --- | --- | --- |
| 1. Was the research question or objective in this paper clearly stated? | Y |  |  |
| 2. Was the study population clearly specified and defined?  🡺 Admissions for exacerbation HF [though not stated first or subsequent] | Y |  |  |
| 3. Was the participation rate of eligible persons at least 50%?  🡺 F. 1: 136 pts = 37% excluded for LVEF < 50%, missing heart echo data, and loss to f/u. | Y |  |  |
| 4. Were all the subjects selected or recruited from the same or similar populations (including the same time period)? Were inclusion and exclusion criteria for being in the study prespecified and applied uniformly to all participants? | Y |  |  |
| 5. Was a sample size justification, power description, or variance and effect estimates provided? |  | N |  |
| 6. For the analyses in this paper, were the exposure(s) of interest measured prior to the outcome(s) being measured? | Y |  |  |
| 7. Was the timeframe sufficient so that one could reasonably expect to see an association between exposure and outcome if it existed?  🡺 Recruitment 2017-2019 [flow chart], f/u 1 year ended 31/12/2020. | Y |  |  |
| 8. For exposures that can vary in amount or level, did the study examine different levels of the exposure as related to the outcome (e.g., categories of exposure, or exposure measured as continuous variable)? | Y |  |  |
| 9. Were the exposure measures (independent variables) clearly defined, valid, reliable, and implemented consistently across all study participants?  🡺 no, “Laboratory data were obtained from the clinical chemistry department within 1 week before discharge” .. that’s rather vague, depending on average length of stay. |  | N |  |
| 10. Was the exposure(s) assessed more than once over time? |  | N |  |
| 11. Were the outcome measures (dependent variables) clearly defined, valid, reliable, and implemented consistently across all study participants?  🡺 within 1 week before d/c – one hopes this is when patients were stabilised on medication, so that HF / inc. volume did not play a large role; but what about extended hospital stays? | Y |  |  |
| 12. Were the outcome assessors blinded to the exposure status of participants? |  |  | NR |
| 13. Was loss to follow-up after baseline 20% or less?  🡺 Lost to f/u were excluded, NR how many. search loss/t no results. Fig. 1: 136 pts = 37% excluded for LVEF < 50%, missing heart echo data, and loss to f/u. |  |  | NR |
| 14. Were key potential confounding variables measured and adjusted statistically for their impact on the relationship between exposure(s) and outcome(s)?  Statin use was NS between groups, but in group with CSR [Cheyne Stokes Resp], those with malnutrition had double the statins as those without. | Y |  |  |

*CD, cannot determine; NA, not applicable; NR, not reported

| Quality rating (Good/ Fair/ Poor) | Poor |
| --- | --- |
| Rater 1 initials: | Fair |
| Rater 2 initials: | Poor |
| Additional comments, if Poor – why? | They have excluded patients lost to f/u, without saying how many. |

## 05. Ikeya 2021 Nihon University Hospital, Tokyo

|  | Yes | No | Other (CD, NR, NA) |
| --- | --- | --- | --- |
| 1. Was the research question or objective in this paper clearly stated?  🡺 Rel/s between severity of malnutrition and clinical outcomes in HF pts receiving cardiac resynchronisation therapy | Y |  |  |
| 2. Was the study population clearly specified and defined?  23/285 excluded for lack of data re. >=1 of the components of CONUT -> 263 pts included [285 – 23 = 262] | Y |  |  |
| 3. Was the participation rate of eligible persons at least 50%? | Y |  |  |
| 4. Were all the subjects selected or recruited from the same or similar populations (including the same time period)? Were inclusion and exclusion criteria for being in the study prespecified and applied uniformly to all participants? | Y |  |  |
| 5. Was a sample size justification, power description, or variance and effect estimates provided?  🡺 Limitations: relatively small sample size [no calculation or estimate] |  | N |  |
| 6. For the analyses in this paper, were the exposure(s) of interest measured prior to the outcome(s) being measured? | Y |  |  |
| 7. Was the timeframe sufficient so that one could reasonably expect to see an association between exposure and outcome if it existed?  🡺 Results, Nutrional status & clin. outcome: median f/u 31 months [10-67] – so some a rather shorter f/u. This is months. OK. And 103/263 died.  Methods: inclusion of patients who underwent CRT device implantation March 2004-October 2020 – and publication submitted May 2021 .. so some had only 6 months f/u. | Y |  |  |
| 8. For exposures that can vary in amount or level, did the study examine different levels of the exposure as related to the outcome (e.g., categories of exposure, or exposure measured as continuous variable)? | Y |  |  |
| 9. Were the exposure measures (independent variables) clearly defined, valid, reliable, and implemented consistently across all study participants? | Y |  |  |
| 10. Was the exposure(s) assessed more than once over time? |  | N |  |
| 11. Were the outcome measures (dependent variables) clearly defined, valid, reliable, and implemented consistently across all study participants?  🡺 No mention of loss to f/u, review of hospital records only -> we don’t know about admission/ death outside of the hospital |  | N |  |
| 12. Were the outcome assessors blinded to the exposure status of participants?  🡺 In methods, F/u & endpoint 🡺 first paper that says reviewers of outcome were blinded. | Y |  |  |
| 13. Was loss to follow-up after baseline 20% or less?  🡺 They say outcomes were reviewed from hospital records. Did records always get notified of someone’s death? |  |  | NR |
| 14. Were key potential confounding variables measured and adjusted statistically for their impact on the relationship between exposure(s) and outcome(s)?  🡺 They adjusted for statin use | Y |  |  |

*CD, cannot determine; NA, not applicable; NR, not reported

| Quality rating (Good/ Fair/ Poor) | Good |
| --- | --- |
| Rater 1 initials: | Good |
| Rater 2 initials: WR | Fair |
| Additional comments, if Poor – why? | F/u measures were **blinded**, but hospital records reviewed only. They **did adjust for statin use.**  There is a miscalculation in Methods, Pts & study protocol [285 reviewed, 23 excluded, 263 included]. Results say 103 out of 263 included died. But no statement re. loss to f/u an only hospital records reviewed. |

## 06. Jia 2023 [Qilu Hospital, Shandong University – province in East China, South of Beijing]

|  | Yes | No | Other (CD, NR, NA) |
| --- | --- | --- | --- |
| 1. Was the research question or objective in this paper clearly stated? |  | N |  |
| 2. Was the study population clearly specified and defined?  🡺 [1] It seems they took any admissions for HF [as long as >= 3 months duration]; No indication of how many people did not match inclusion criteria/ lost to f/u |  | N |  |
| 3. Was the participation rate of eligible persons at least 50%?  🡺 retrospective .. they just took all those who fulfilled inclusion crit. |  |  | NA |
| 4. Were all the subjects selected or recruited from the same or similar populations (including the same time period)? Were inclusion and exclusion criteria for being in the study prespecified and applied uniformly to all participants?  🡺 Methods: included = Admissions for HF Jan 2017 – May 2018 [500] + Sx and signs + At least one **auxiliary examination** ***such as*** heart echo or NT-pro-BNP [WR: i.e. – what else?] + duration of disease >= 3 months. Exclusion age < 18 years, d/c or death < 24 ours of admission.  Results, 1^st^ para: among the **auxiliary examinations** to assess the conditions of patients .. ECG [under tables defined as electrocardiogram] accounted for the highest proportion.  I feel this is too vague: ‘At least one .. such as’. Possibly a language problem. |  | N |  |
| 5. Was a sample size justification, power description, or variance and effect estimates provided? |  | N |  |
| 6. For the analyses in this paper, were the exposure(s) of interest measured prior to the outcome(s) being measured? | Y |  |  |
| 7. Was the timeframe sufficient so that one could reasonably expect to see an association between exposure and outcome if it existed?  🡺 No, condition on admission vs. on d/c .. they don’t say when outcome measures were taken before d/c .. did they do another echo or BNP before d/c, in those who did not die/ go to ICU/ self-dc due to deteriorating condition. |  | N |  |
| 8. For exposures that can vary in amount or level, did the study examine different levels of the exposure as related to the outcome (e.g., categories of exposure, or exposure measured as continuous variable)? | Y |  |  |
| 9. Were the exposure measures (independent variables) clearly defined, valid, reliable, and implemented consistently across all study participants?  🡺 Let’s assume this one is a yes, though again, lab methods not described. | Y |  |  |
| 10. Was the exposure(s) assessed more than once over time?  🡺 not stated. |  | N |  |
| 11. Were the outcome measures (dependent variables) clearly defined, valid, reliable, and implemented consistently across all study participants?  🡺 Deterioration vs. improvement groups.. what about no change? or some deterioration and some improvement? How was improvement defined [not stated].. what about those whose HF was diagnosed only with ECG – how was their improvement measured?.. They do report EF on it seems all patients [p. 08-09, preserved or reduced EF]  Measured within the 1 admission – with admissions > 24 hours included. Does not allow for intercurrent conditions.  🡺 In Results/ prognosis, all of a sudden they state “The readmission interval represents the frequency of patient’s deterioration in a period of time ..”  WR:   - for frequency you’d have to record admissions over a longer period of time. - They state nothing about f/u time |  | N |  |
| 12. Were the outcome assessors blinded to the exposure status of participants? |  |  | NR |
| 13. Was loss to follow-up after baseline 20% or less?  🡺 No statement about loss to follow-up. Search on ‘los’ [covering loss and lost] – nothing. Search for follow -> only under limitations “lack of f/u data for out-of-hospital tx” |  |  | NR |
| 14. Were key potential confounding variables measured and adjusted statistically for their impact on the relationship between exposure(s) and outcome(s)?  🡺 Statistical methods poorly described |  |  | CD |

*CD, cannot determine; NA, not applicable; NR, not reported

| Quality rating (Good/ Fair/ Poor) | Poor |
| --- | --- |
| Rater 1 initials: | Poor |
| Rater 2 initials: | Poor |
| Additional comments, if Poor – why? | Poorly written, in places hard to tell what they mean. Aim not clearly stated. Methods appear half in Results [i.e. rehospitalisation interval], not clearly stated what they regard as improvement or not – while main outcome measure seems to be improvement/ deterioration while in hospital. No statement re. exclusion due to lack of data, or loss to f/u. |

## 07. Kinugasa 2022 – Tottori University, West Japan

|  | Yes | No | Other (CD, NR, NA) |
| --- | --- | --- | --- |
| 1. Was the research question or objective in this paper clearly stated?  🡺 Good: they start abstract with aim and end introduction with it. Though what they mean by different measures [e.g. ‘physical function’ was not easy to find out] | Y |  |  |
| 2. Was the study population clearly specified and defined?  🡺 First one that recruited prospectively, in outpatients, pts > 1 month in outpatients, >=1 admission due to HF in last 5 years. Though still people at varying severity of HF, presumably.  Recruited from OPD Dec 2012-Sept 2014. | Y |  |  |
| 3. Was the participation rate of eligible persons at least 50%?  🡺 Prospective study, they say under limitations [p. 10/11] that patient selection bias [for milder HF, class I-II] must be considered. |  |  | NR |
| 4. Were all the subjects selected or recruited from the same or similar populations (including the same time period)? Were inclusion and exclusion criteria for being in the study prespecified and applied uniformly to all participants?  🡺 Though again, severity/ duration of HF not recorded, except all had had 1 admission in past 5 years. That’s an attempt at creating a more homogeneous sample. | Y |  |  |
| 5. Was a **sample** size justification, **power** description, or **variance** and effect estimates provided? & **calc**  **🡺 searches for these words - nothing** |  | N |  |
| 6. For the analyses in this paper, were the exposure(s) of interest measured prior to the outcome(s) being measured?  🡺 Assume yes, as prospective prognostic study, but not stated explicitly. | Y |  |  |
| 7. Was the timeframe sufficient so that one could reasonably expect to see an association between exposure and outcome if it existed?  🡺 Methods, patients: f/u 1 year. | Y |  |  |
| 8. For exposures that can vary in amount or level, did the study examine different levels of the exposure as related to the outcome (e.g., categories of exposure, or exposure measured as continuous variable)? |  | N |  |
| 9. Were the exposure measures (independent variables) clearly defined, valid, reliable, and implemented consistently across all study participants? | Y |  |  |
| 10. Was the exposure(s) assessed more than once over time? |  | N |  |
| 11. Were the outcome measures (dependent variables) clearly defined, valid, reliable, and implemented consistently across all study participants?  LV-EF – not explained how they measured.  They do not explain how they retrieved re-admission & mortality data – only in the same hospital, or elsewhere? |  | N |  |
| 12. Were the outcome assessors blinded to the exposure status of participants?  From how the study was set up, it seems likely that they did all the measurements in one visit. However, the research nurses did not necessarily have access to |  |  | NR |
| 13. Was loss to follow-up after baseline 20% or less?  🡺 No statement about loss to f/u. |  |  | NR |
| 14. Were key potential confounding variables measured and adjusted statistically for their impact on the relationship between exposure(s) and outcome(s)?  🡺 They mention the effect of statins; And have evaluated AUC, physical function, and prognosis by statin use. | Y |  |  |

*CD, cannot determine; NA, not applicable; NR, not reported

| Quality rating (Good/ Fair/ Poor) | Fair |
| --- | --- |
| Rater 1 initials: | Fair |
| Rater 2 initials: | Fair |
| Additional comments, if Poor – why? | It seems a solid study, multi-centre, prospective. I suspect they had a large dataset and wanted some more publications out of it. What was prospectively researched?  No statement re. loss t f/u. As prospective study this was likely more limited, but still, should have been reported.  They have adjusted for statin use. |

## 08. La Rovere 2017 [Montanesco, Italy]

|  | Yes | No | Other (CD, NR, NA) |
| --- | --- | --- | --- |
| 1. Was the research question or objective in this paper clearly stated? | Y |  |  |
| 2. Was the study population clearly specified and defined?  🡺 All admitted with HF who could complete 6MW [6 min. walking test] | Y |  |  |
| 3. Was the participation rate of eligible persons at least 50%?  🡺 466 out of 533 .. and an exact account of why the 67 could not do the 6MWT. | Y |  |  |
| 4. Were all the subjects selected or recruited from the same or similar populations (including the same time period)? Were inclusion and exclusion criteria for being in the study prespecified and applied uniformly to all participants?  🡺 All admitted pts to HF unit Jan 2008-Dec 2010 – 118 for decompensation, rest for optimising of Tx, evaluation, re-assessment of indication for heart transplant | Y |  |  |
| 5. Was a sample size justification, power description, or variance and effect estimates provided? |  | N |  |
| 6. For the analyses in this paper, were the exposure(s) of interest measured prior to the outcome(s) being measured? | Y |  |  |
| 7. Was the timeframe sufficient so that one could reasonably expect to see an association between exposure and outcome if it existed? | Y |  |  |
| 8. For exposures that can vary in amount or level, did the study examine different levels of the exposure as related to the outcome (e.g., categories of exposure, or exposure measured as continuous variable)?  🡺 CONUT cutoff 0-1 and >1. |  | N |  |
| 9. Were the exposure measures (independent variables) clearly defined, valid, reliable, and implemented consistently across all study participants? | Y |  |  |
| 10. Was the exposure(s) assessed more than once over time? |  | N |  |
| 11. Were the outcome measures (dependent variables) clearly defined, valid, reliable, and implemented consistently across all study participants?  🡺 Yes, and they even investigated patients’ whereabouts with referring physician and relatives. | Y |  |  |
| 12. Were the outcome assessors blinded to the exposure status of participants?  🡺 search on blind no results. Depends on whether the 6MWT was done by nurses who had access to lab results AND were able to interpret them, or by research assistants blinded to everything else. |  |  | NR |
| 13. Was loss to follow-up after baseline 20% or less?  🡺 none were lost | Y |  |  |
| 14. Were key potential confounding variables measured and adjusted statistically for their impact on the relationship between exposure(s) and outcome(s)?  🡺 Not adjusted for statins. |  | N |  |

*CD, cannot determine; NA, not applicable; NR, not reported

| Quality rating (Good/ Fair/ Poor) | Good |
| --- | --- |
| Rater 1 initials: | Good |
| Rater 2 initials: | Fair |
| Additional comments, if Poor – why? | **Very clearly written**, have investigated [cause of] death with referring physician & relatives. Discuss all exclusions & loss to f/u [none]. Not adjusted for statin use, statins not even mentioned.  Would be good if they’d adjusted for statin use or at least discussed it. |

## 09. Nishi 2017 – [Tsuchiura Japan]

|  | Yes | No | Other (CD, NR, NA) |
| --- | --- | --- | --- |
| 1. Was the research question or objective in this paper clearly stated?   🡺 First thing in abstract | Y |  |  |
| 2. Was the study population clearly specified and defined? | Y |  |  |
| 3. Was the participation rate of eligible persons at least 50%?  🡺 AND they checked that baseline characteristics and mortality in excluded patients did not differ from those in incuded patients.  Excluded: if 2-year observation predicted to be impossible  Excluded: from 838 -> 356 for missing data = > 42%  Prospective: written informed consent obtained | Y |  |  |
| 4. Were all the subjects selected or recruited from the same or similar populations (including the same time period)? Were inclusion and exclusion criteria for being in the study prespecified and applied uniformly to all participants?  🡺 Hospitalized June 2012-March 2015 | Y |  |  |
| 5. Was a sample size justification, power description, or variance and effect estimates provided?  🡺 All searches nil result |  | N |  |
| 6. For the analyses in this paper, were the exposure(s) of interest measured prior to the outcome(s) being measured?  🡺 Yes. < 72 hrs of admission [p 3/13, Data collection] | Y |  |  |
| 7. Was the timeframe sufficient so that one could reasonably expect to see an association between exposure and outcome if it existed? | Y |  |  |
| 8. For exposures that can vary in amount or level, did the study examine different levels of the exposure as related to the outcome (e.g., categories of exposure, or exposure measured as continuous variable)? | Y |  |  |
| 9. Were the exposure measures (independent variables) clearly defined, valid, reliable, and implemented consistently across all study participants? | Y |  |  |
| 10. Was the exposure(s) assessed more than once over time?  🡺 Bottom p. 11/13, under limitations: CONUT only assessed on admission. |  | N |  |
| 11. Were the outcome measures (dependent variables) clearly defined, valid, reliable, and implemented consistently across all study participants?  🡺 This is their primary outcome, and they do not say how they assessed it. hospital records only .. but they enrolled prospectively, with consent, so easier to keep track of people. |  |  | NR |
| 12. Were the outcome assessors blinded to the exposure status of participants? |  |  | NR |
| 13. Was loss to follow-up after baseline 20% or less? |  |  | NR |
| 14. Were key potential confounding variables measured and adjusted statistically for their impact on the relationship between exposure(s) and outcome(s)?  🡺 not adjusted for statins. U  🡺 Under limitations: ‘we did not exclude comorbid diseases such as nephrotic syndrome, infectious diseases, and blood disorders, which can affect ..[albumin, cholesterol, lymphocyte count]. |  | N |  |

*CD, cannot determine; NA, not applicable; NR, not reported

| Quality rating (Good/ Fair/ Poor) | Fair |
| --- | --- |
| Rater 1 initials: | Fair |
| Rater 2 initials: | Fair |
| Additional comments, if Poor – why? | On the whole it reads well. However, not reporting how they assessed their primary outcome [death] or whether assessors were blinded, or whether any were lost to f/u.  Not adjusted for statin use, not excluded nephrotic sy/ infect. dis/ blood disorders.  > 42% lost to f/u re. missing data. They DID check that baseline characteristics of missing data patients were comparable to those of enrolled patients, AND mortality not more frequent in excluded than included patients -> that earns them ‘fair’. |

## 10. Rubio-Garcia 2021 – Zaragoza, Spain

|  | Yes | No | Other (CD, NR, NA) |
| --- | --- | --- | --- |
| 1. Was the research question or objective in this paper clearly stated?  🡺 end of introduction | Y |  |  |
| 2. Was the study population clearly specified and defined?  🡺 Admissions HF + accepting f/u in specialised clinic.  Exclusions: previous admission ITU, renal replacement Tx, sign. valvular disease, advanced COPD, acute HF due to arrhythmia. | Y |  |  |
| 3. Was the participation rate of eligible persons at least 50%?  P. 373 top right: “No imputations were made for losses since only patients with all study variables registered in the database were selected. |  |  | NR |
| 4. Were all the subjects selected or recruited from the same or similar populations (including the same time period)? Were inclusion and exclusion criteria for being in the study prespecified and applied uniformly to all participants? | y |  |  |
| 5. Was a sample size justification, power description, or variance and effect estimates provided? |  | N |  |
| 6. For the analyses in this paper, were the exposure(s) of interest measured prior to the outcome(s) being measured?  🡺 top of p 3/9: on admission. | Y |  |  |
| 7. Was the timeframe sufficient so that one could reasonably expect to see an association between exposure and outcome if it existed?  🡺 p. 6/9, “During the year following admission” | Y |  |  |
| 8. For exposures that can vary in amount or level, did the study examine different levels of the exposure as related to the outcome (e.g., categories of exposure, or exposure measured as continuous variable)?  🡺 Survival curves acc. to N, mild, mod, severe. | Y |  |  |
| 9. Were the exposure measures (independent variables) clearly defined, valid, reliable, and implemented consistently across all study participants?  🡺 Blood tests on admission [p. 3, under Nutritional status analysis, and under Congestion makers | Y |  |  |
| 10. Was the exposure(s) assessed more than once over time? | N |  |  |
| 11. Were the outcome measures (dependent variables) clearly defined, valid, reliable, and implemented consistently across all study participants?  🡺 not reported |  |  | CD |
| 12. Were the outcome assessors blinded to the exposure status of participants? |  |  | NR |
| 13. Was loss to follow-up after baseline 20% or less?  See Q. 3 🡺 Top of p. 3/9: “No imputations were made for losses since only those patients with all study variables registered in the database were selected.” |  |  | CD |
| 14. Were key potential confounding variables measured and adjusted statistically for their impact on the relationship between exposure(s) and outcome(s)? | Y |  |  |

*CD, cannot determine; NA, not applicable; NR, not reported

| Quality rating (Good/ Fair/ Poor) |  |
| --- | --- |
| Rater 1 initials: | Fair |
| Rater 2 initials: | Poor |
| Additional comments, if Poor – why? | Only patients selected for whom they had all variables.. this may not affect our measurements, but as a cohort study looking at prognostic value of malnutrition for all-cause mortality, that sounds like poor study design to me. Didn’t the statistician/ peer reviewers not pick up on this? They should at least have reported how many had missing data. |

## 11. Shirakabe 2018 [Chiba, Japan - peninsula S-W of Tokyo]

|  | Yes | No | Other (CD, NR, NA) |
| --- | --- | --- | --- |
| 1. Was the research question or objective in this paper clearly stated?  🡺 End of introduction is vague: prognostic efficacy of PNI and CONUT .. malnutrition .. as predictor of adverse outcomes in pts with acute HF. 🡪 I find this rather vague .. what kind of prognosis/ adverse outcomes? No clear hypothesis to test. In Methods, short-term prognosis, duration of ICU & hospital stay, in-hospital mortality => i.e. a very short-term prognosis. |  | N |  |
| 2. Was the study population clearly specified and defined?  🡺 ITU admission for HF in Chira hospital | Y |  |  |
| 3. Was the participation rate of eligible persons at least 50%?  🡺 Exclusion if not all data available from records, and if blood sample not < 30 min of hopsitalisation  1214 patients admitted Jan 2000-Jun 2016, exclusions:   - 211 pts re. missing ly/ cholesterol - 462 pts who had bloods > 1 day after admission. - 83 whose bloods not taken < 30 min of admission. - == 458 left. |  | N |  |
| 4. Were all the subjects selected or recruited from the same or similar populations (including the same time period)? Were inclusion and exclusion criteria for being in the study prespecified and applied uniformly to all participants? | Y |  |  |
| 5. Was a sample size justification, power description, or variance and effect estimates provided?  🡺 Under limitations, sample size was relatively low. searches for sample, size, estimate, calculat*, power, variance – nil result. |  | N |  |
| 6. For the analyses in this paper, were the exposure(s) of interest measured prior to the outcome(s) being measured?  🡺 Blood samples < 30 min of admission | Y |  |  |
| 7. Was the timeframe sufficient so that one could reasonably expect to see an association between exposure and outcome if it existed?  🡺 Methods only mentions in-hospital mortality, length of stay [LOS] in ICU, LOS in hospital. But the Kaplan-Meier curve p 9/11 shows? what on the X axis? Only in Discussion do they talk about long-term prognosis < 365 days | Y |  |  |
| 8. For exposures that can vary in amount or level, did the study examine different levels of the exposure as related to the outcome (e.g., categories of exposure, or exposure measured as continuous variable)? | Y |  |  |
| 9. Were the exposure measures (independent variables) clearly defined, valid, reliable, and implemented consistently across all study participants?  🡺 Yes. All definitions and calculation methods are given. | Y |  |  |
| 10. Was the exposure(s) assessed more than once over time? |  | N |  |
| 11. Were the outcome measures (dependent variables) clearly defined, valid, reliable, and implemented consistently across all study participants?  🡺 Though they might have mentioned how they calculated in-hospital/ ICU length of stay. They did pick up phone to enquire about outcome at 1 year. | Y |  |  |
| 12. Were the outcome assessors blinded to the exposure status of participants? |  |  | NR |
| 13. Was loss to follow-up after baseline 20% or less?  🡺 search for loss/ lost/follow – nil. |  |  | NR |
| 14. Were key potential confounding variables measured and adjusted statistically for their impact on the relationship between exposure(s) and outcome(s)?  🡺 Statins are named in discussion, but no mention they were adjusted for. |  | N |  |

*CD, cannot determine; NA, not applicable; NR, not reported

| Quality rating (Good/ Fair/ Poor) | Poor |
| --- | --- |
| Rater 1 initials: | Fair |
| Rater 2 initials: | Poor |
| Additional comments, if Poor – why? | Aim is somewhat vague, no statement of primary outcome.  Lots of people were excluded because bloods not done < 30 min of admission. Unlikely that they wanted to exclude haemodilution from iv fluids, as there’s fluid overload already. Not stated they adjusted for statin use.  They had such strict criteria for bloods < 30 min. of admission, that they included < 50% of eligible patients. |

## 12. Takada 2021 [Tokyo Women’s Medical University Hospital]

|  | Yes | No | Other (CD, NR, NA) |
| --- | --- | --- | --- |
| 1. Was the research question or objective in this paper clearly stated? | Y |  |  |
| 2. Was the study population clearly specified and defined? | Y |  |  |
| 3. Was the participation rate of eligible persons at least 50%?  Excluded: missing data, lost to f/u  Eligible: 1930 pts d/c alive, 180 missing CONUT scores, 45 lost to f/u. = total of 11.7% excluded. | Y |  |  |
| 4. Were all the subjects selected or recruited from the same or similar populations (including the same time period)? Were inclusion and exclusion criteria for being in the study prespecified and applied uniformly to all participants?  🡺 all admitted for HF to Tokyo Women’s med univ hospital July 2013 – Sept 2019. | Y |  |  |
| 5. Was a sample size justification, power description, or variance and effect estimates provided?  🡺 searches sample/ size/ power/ variance/ estimat* - nil |  | N |  |
| 6. For the analyses in this paper, were the exposure(s) of interest measured prior to the outcome(s) being measured? | Y |  |  |
| 7. Was the timeframe sufficient so that one could reasonably expect to see an association between exposure and outcome if it existed? | Y |  |  |
| 8. For exposures that can vary in amount or level, did the study examine different levels of the exposure as related to the outcome (e.g., categories of exposure, or exposure measured as continuous variable)? |  | N |  |
| 9. Were the exposure measures (independent variables) clearly defined, valid, reliable, and implemented consistently across all study participants? | Y |  |  |
| 10. Was the exposure(s) assessed more than once over time? |  | N |  |
| 11. Were the outcome measures (dependent variables) clearly defined, valid, reliable, and implemented consistently across all study participants? | Y |  |  |
| 12. Were the outcome assessors blinded to the exposure status of participants? |  |  | NR |
| 13. Was loss to follow-up after baseline 20% or less?  🡺 P 3/11: Results, Study population: Eligible: 1930 pts d/c alive, 180 missing CONUT scores, 45 lost to f/u. = total of 11.7% excluded. 🡺 1705 analysed. | Y |  |  |
| 14. Were key potential confounding variables measured and adjusted statistically for their impact on the relationship between exposure(s) and outcome(s)?  🡺 Top left p. 3/11 multivariate analysis was adjusted for a number of meds, **but not for statins**  🡺 P 6/11 bottom left: no statin use independently associated with normalisation of CONUT scores at d/c. |  | N |  |

*CD, cannot determine; NA, not applicable; NR, not reported

| Quality rating (Good/ Fair/ Poor) | Fair |
| --- | --- |
| Rater 1 initials: | Fair |
| Rater 2 initials: | Fair |
| Additional comments, if Poor – why? | Good accounting of numbers for missing data & loss to f/u.  Not adjusted for statin use – **even though they give a statistically significant difference between groups on basis of statin use before admission** [Table 1]. |

## 13. Uemura 2022 Anjo [Kosei Hospital, Japan .. W of Tokyo, just N of the bay W of Hamatsu]

|  | Yes | No | Other (CD, NR, NA) |
| --- | --- | --- | --- |
| 1. Was the **research question** or objective in this paper clearly stated? | Y |  |  |
| 2. Was the **study population** clearly specified and defined?  🡺 Adm HF 2016-2018, Anjo Kosei Hospital | Y |  |  |
| 3. Was the **participation rate** of eligible persons at least 50%?  🡺 Eligible 713, exc. in-hospital death, lack of data -> study population 465. | Y |  |  |
| 4. Were **all the subjects selected or recruited from the same or similar populations** (including the same time period)? Were inclusion and exclusion criteria for being in the study prespecified and applied uniformly to all participants? | Y |  |  |
| 5. Was a **sample size** justification, power description, or variance and effect estimates provided?  🡺 Search for sample/ size/ power/calcul*/variance/estimate -> nil |  | N |  |
| 6. For the analyses in this paper, were the **exposure**(s) of interest **measured prior to the outcome**(s) being measured? | Y |  |  |
| 7. Was the **timeframe sufficient** so that one could reasonably expect to see an association between exposure and outcome if it existed?  🡺 Methods, f/u & assessment: up to 1 year after d/c | Y |  |  |
| 8. For exposures that can vary in amount or level, did the study examine **different levels of the exposure** as related to the outcome (e.g., categories of exposure, or exposure measured as continuous variable)?  🡺 CONUT normal, mild, severe, same for GNRI, MNA-SF. [see Kaplan-Meier curves] | Y |  |  |
| 9. Were the **exposure measures** (independent variables) clearly defined, valid, reliable, and implemented consistently across all study participants?  🡺 They even mention lab methods. | Y |  |  |
| 10. Was the **exposure(s) assessed more than once** over time? |  | N |  |
| 11. Were the **outcome measures** (dependent variables) clearly defined, valid, reliable, and implemented consistently across all study participants?  🡺 Assessed by experienced cardiologists. That leaves some room for subjectivity, but I suppose there always is? | Y |  |  |
| 12. Were the **outcome assessors blinded** to the exposure status of participants?  🡺 Methods, F/u & assessments: Assessed by experienced cardiologists. No indication of blinding. |  |  | NR |
| 13. Was **loss to follow-up** **after baseline 20% or less?**  Eligible 713 – 67 = 646  Missing data n=168 = 26%  No mention of loss to f/u after d/c. |  | N |  |
| 14. Were key potential **confounding variables** measured and **adjusted statistically** for their impact on the relationship between exposure(s) and outcome(s)?  🡺 Methods, Statistical analyses: after adjustment for variables with P-values < 0.05 in univariate analysis. | Y |  |  |

*CD, cannot determine; NA, not applicable; NR, not reported

| Quality rating (Good/ Fair/ Poor) | Good |
| --- | --- |
| Rater 1 initials: | Good |
| Rater 2 initials: | Fair |
| Additional comments, if Poor – why? | Good accounting of all missing data, but no mention of poss. loss to f/u.  I take in-hospital death as a genuine exclusion criterium – i.e. those are not lost to f/u. Still 26% missing data. |

## 14. Zhao 2023 [Second Hospital of Dalian Medical Univ, Dalian, Liaoning, China]

|  | Yes | No | Other (CD, NR, NA) |
| --- | --- | --- | --- |
| 1. Was the **research question** or objective in this paper clearly stated? | Y |  |  |
| 2. Was the **study population** clearly specified and defined?  🡺 187 systolic HF case hospitalised Jan 2016-July 2021; see Fig 1 | Y |  |  |
| 1. Was the **participation rate** of eligible persons at least 50%? 2. See Q 13 | Y |  |  |
| 4. Were **all the subjects selected or recruited from the same or similar populations** (including the same time period)? Were inclusion and exclusion criteria for being in the study prespecified and applied uniformly to all participants? | Y |  |  |
| 5. Was a **sample size** justification, power description, or variance and effect estimates provided?  🡺 Search for sample/ size/ power/calcul*/variance/estimate – nil.  They do admit their sample size is small. |  | N |  |
| 6. For the analyses in this paper, were the **exposure**(s) of interest **measured prior to the outcome**(s) being measured? | Y |  |  |
| 7. Was the **timeframe sufficient** so that one could reasonably expect to see an association between exposure and outcome if it existed?  🡺 1ary endpoint = all-cause death in first 90 days after d/c .. but they have found significant differences between groups, even with 3 months f/u |  | N |  |
| 8. For exposures that can vary in amount or level, did the study examine **different levels of the exposure** as related to the outcome (e.g., categories of exposure, or exposure measured as continuous variable)? | Y |  |  |
| 9. Were the **exposure measures** (independent variables) clearly defined, valid, reliable, and implemented consistently across all study participants? | Y |  |  |
| 10. Was the **exposure(s) assessed more than once** over time? |  | N |  |
| 11. Were the **outcome measures** (dependent variables) clearly defined, valid, reliable, and implemented consistently across all study participants?  🡺 1ary = all-cause mortality, 2ary = re-admission | Y |  |  |
| 12. Were the **outcome assessors blinded** to the exposure status of participants? |  |  | NR |
| 13. Was **loss to follow-up** **after baseline 20% or less?**  **🡺 Fig. 1:** Of original 208 pts who met eligibility criteria, 8 absence of CONUT data, 13 lost to f/u == just over 10% | Y |  |  |
| 14. Were key potential **confounding variables** measured and **adjusted statistically** for their impact on the relationship between exposure(s) and outcome(s)?  🡺 Yes, and they looked at statins too .. more people in normal nutrition used statins than in malnutrition groups. | Y |  |  |

*CD, cannot determine; NA, not applicable; NR, not reported

| Quality rating (Good/ Fair/ Poor) | Good |
| --- | --- |
| Rater 1 initials: | Good |
| Rater 2 initials: | Good |
| Additional comments, if Poor – why? |  |

**Table S3.** Risk of bias assessment using GNRI scores.

| Study  Year | Q1 | Q2 | Q3 | Q4 | Q5 | Q6 | Q7 | Q8 | Q9 | Q10 | Q11 | Q12 | Q13 | Q14 | Overall |
| --- | --- | --- | --- | --- | --- | --- | --- | --- | --- | --- | --- | --- | --- | --- | --- |
| Hirose 2020 | Y | Y | Y | Y | N | Y | Y | Y | Y | N | N | N | Y | Y | Good |
| Hirose 2021 | Y | Y | Y | Y | N | Y | Y | N | Y | N | Y | NR | N | Y | Poor |
| Honda 2016 | Y | Y | Y | Y | N | Y | N | N | Y | N | Y | NR | CD | Y | Fair |
| Kawakubo 2022 | Y | Y | Y | Y | N | Y | Y | Y | Y | N | NR | Y | N | Y | Good |
| Kinugasa 2013 | Y | Y | Y | Y | N | Y | Y | Y | Y | N | Y | NR | Y | Y | Fair |
| Kitamura 2019 | Y | Y | Y | Y | N | NR | Y | N | Y | N | Y | NR | Y | Y | Good |
| Nishi 2019 | Y | Y | Y | Y | N | Y | Y | N | Y | N | N | NR | N | Y | Poor |
| Sargento 2017 | Y | Y | Y | Y | N | Y | Y | Y | Y | N | Y | NR | Y | Y | Good |
| Sze 2019 | Y | Y | NR | Y | N | Y | Y | Y | NR | N | Y | NR | CD | Y | Fair |
| Yasumura 2020 | Y | Y | CD | Y | N | Y | Y | Y | Y | NR | NR | NR | CD | Y | Fair |

## 15. Hirose 2020 [Juntendo Univ. Hospital, Tokyo]

|  | Yes | No | Other (CD, NR, NA) |
| --- | --- | --- | --- |
| 1. Was the **research question** or objective in this paper clearly stated?  Effect of GNR & other risk factors on LOS in pts admitted to ICU for HF | Y |  |  |
| 2. Was the **study population** clearly specified and defined?  Materials, subjects: Pts admitted to cardiac ICU for acute decompensated HF Jan 2007 - Dec 2011 | Y |  |  |
| 3. Was the **participation rate** of eligible persons at least 50%?  751 pts admitted. 190 excluded re. ACS, malignancy, surgery, long-term haemodialysis == 561 eligible. 110 had missing data – excluded. == 19.6% | Y |  |  |
| 4. Were **all the subjects selected or recruited from the same or similar populations** (including the same time period)? Were inclusion and exclusion criteria for being in the study prespecified and applied uniformly to all participants? | Y |  |  |
| 5. Was a **sample size** justification, power description, or variance and effect estimates provided?  🡺 Search for sample/ size/ power/calcul*/variance/estimate - nil |  | N |  |
| 6. For the analyses in this paper, were the **exposure**(s) of interest **measured prior to the outcome**(s) being measured?  On admission | Y |  |  |
| 7. Was the **timeframe sufficient** so that one could reasonably expect to see an association between exposure and outcome if it existed? | Y |  |  |
| 8. For exposures that can vary in amount or level, did the study examine **different levels of the exposure** as related to the outcome (e.g., categories of exposure, or exposure measured as continuous variable)? | Y |  |  |
| 9. Were the **exposure measures** (independent variables) clearly defined, valid, reliable, and implemented consistently across all study participants?  BMI was obtained on admission – in patients sick enough to need admission? can they stand on the scales [or were these the patients excluded for missing data], and how much of that weight is fluid overload? .. though presumably they all had some extra weight due to fluid overload. | Y |  |  |
| 10. Was the **exposure(s) assessed more than once** over time? | N |  |  |
| 11. Were the **outcome measures** (dependent variables) clearly defined, valid, reliable, and implemented consistently across all study participants?  LOS is difficult, esp. if some stay longer for social reasons [as they would in UK]: no place in NH/ waiting for adaptations at home, etc. |  | N |  |
| 12. Were the **outcome assessors blinded** to the exposure status of participants?  Search for “blind” – nil. Doctors who knew GNRI were also responsible for d/c of patient sooner or later, thereby could have influenced LOS. If doctors used the GNRI to keep pts with malnutrition in hospital for longer, this would be a major confounding factor. |  | N |  |
| 13. Was **loss to follow-up** **after baseline 20% or less?**  19.6% had missing data. As f/u was only as long as the hospital stay, no further loss to f/u .. so only just a Yes. | Y |  |  |
| 14. Were key potential **confounding variables** measured and **adjusted statistically** for their impact on the relationship between exposure(s) and outcome(s)?  Yes: first univariate analysis of a long list of variables, then multivariate analysis of all variables with p-value < 0.15 [i.e. quite broad]. | Y |  |  |

*CD, cannot determine; NA, not applicable; NR, not reported

| Quality rating (Good/ Fair/ Poor) | Good |
| --- | --- |
| Rater 1 initials: | Fair |
| Rater 2 initials: | Good |
| Additional comments, if Poor – why? |  |

## 16. Hirose 2021 [Multicentre study, 15 Japanese hospitals]

|  | Yes | No | Other (CD, NR, NA) |
| --- | --- | --- | --- |
| 1. Was the **research question** or objective in this paper clearly stated? | Y |  |  |
| 2. Was the **study population** clearly specified and defined?  Abstract: 890 hospitalised pts >= 65 yo with decomp. HF [reduced or preserved EF], able to ambulate at d/c  Methods, population: **exclusion**: transplant, assist device, dialysis, acute myocarditis; missing BNP/ pro-BNP, and BNP < 100, pro-BNP < 300. | Y |  |  |
| 3. Was the **participation rate** of eligible persons at least 50%?  1332 pts registered in FRAGILE-HF study  261 excluded re. missing data = 19.6%  181 excluded re. implanted cardiac device [BIA not safe] ‘or simply had missing data’ = 13.6%  🡺 890 pts analysed | Y |  |  |
| 4. Were **all the subjects selected or recruited from the same or similar populations** (including the same time period)? Were inclusion and exclusion criteria for being in the study prespecified and applied uniformly to all participants? | Y |  |  |
| 5. Was a **sample size** justification, power description, or variance and effect estimates provided?  🡺 Search for sample/ size/ power/calcul*/variance/estimate – nil. Same search in Matsue [main study] – nil.  **Pts with missing BNP/ pro-BNP were excluded** |  | N |  |
| 6. For the analyses in this paper, were the **exposure**(s) of interest **measured prior to the outcome**(s) being measured? | Y |  |  |
| 7. Was the **timeframe sufficient** so that one could reasonably expect to see an association between exposure and outcome if it existed? | Y |  |  |
| 8. For exposures that can vary in amount or level, did the study examine **different levels of the exposure** as related to the outcome (e.g., categories of exposure, or exposure measured as continuous variable)?  GNRI cut-off 92, GLIM |  | N |  |
| 9. Were the **exposure measures** (independent variables) clearly defined, valid, reliable, and implemented consistently across all study participants?  **MUST** >= 1 = risk  **GLIM**: at least one phenotypic [BMI, weight loss, reduced muscle mass] and one etiological [reduced food intake or absorption; acute disease/ chronic inflammation]  **GNRI**: calculation using albumin and BMI. | Y |  |  |
| 10. Was the **exposure(s) assessed more than once** over time? | N |  |  |
| 11. Were the **outcome measures** (dependent variables) clearly defined, valid, reliable, and implemented consistently across all study participants?  Prim. outcome: all-cause mortality < 1 year of d/c  f/u at OPD, or from medical records of other medical facilities, or from family. | Y |  |  |
| 12. Were the **outcome assessors blinded** to the exposure status of participants? |  |  | NR |
| 13. Was **loss to follow-up** **after baseline 20% or less?**  Results 1^st^ paragraph: 19.6% missing data PLUS 13.6% who had an implanted device ‘or simply had missing data’ 🡺 I wish they had specified how many missing data & how many had implanted device.  Search for lost/ loss/ follow – nil. Total missing data between 19.6 and 33.2%. |  | N |  |
| 14. Were key potential **confounding variables** measured and **adjusted statistically** for their impact on the relationship between exposure(s) and outcome(s)?  Multivariate Cox regression models | Y |  |  |

*CD, cannot determine; NA, not applicable; NR, not reported

| Quality rating (Good/ Fair/ Poor) | Poor |
| --- | --- |
| Rater 1 initials: | Fair |
| Rater 2 initials: | Poor |
| Additional comments, if Poor – why? | Poor because no good accounting of missing data. Esp. lumping some with missing data together with genuine exclusion for implanted device. Otherwise, it would have been ‘good’. Including checking for mortality with other medical facilities and family. |

## 17. Honda 2016 [National Cerebral and Cardiovascular Centre, Osaka, Japan]

|  | Yes | No | Other (CD, NR, NA) |
| --- | --- | --- | --- |
| 1. Was the **research question** or objective in this paper clearly stated? | Y |  |  |
| 2. Was the **study population** clearly specified and defined?  Data from National Cerebral & Cardiovasc. Centre Acute Decompensated Heart Failure [NaDEF] registry, obtained Jan 2013-Mar 2015 == single-centre registry, ongoing prospective cohort of pts >= 20 yo, Dx AHF by at least 2 experienced cardiol. acc to Framingham criteria | Y |  |  |
| 3. Was the **participation rate** of eligible persons at least 50%?  651 pts enrolled in the register  exclusion: those with ACS, < 64 yo, missing GNRI data  🡺 490 pts analysed = 75% | Y |  |  |
| 4. Were **all the subjects selected or recruited from the same or similar populations** (including the same time period)? Were inclusion and exclusion criteria for being in the study prespecified and applied uniformly to all participants? | Y |  |  |
| 5. Was a **sample size** justification, power description, or variance and effect estimates provided?  🡺 Search for sample/ size/ power/calcul*/variance/estimate  Power comes only in recommendations for further research |  | N |  |
| 6. For the analyses in this paper, were the **exposure**(s) of interest **measured prior to the outcome**(s) being measured? | Y |  |  |
| 7. Was the **timeframe sufficient** so that one could reasonably expect to see an association between exposure and outcome if it existed?  Methods: F/u 24 months after d/c. Results: median f/u 189 days [IQR 66-421 days] .. i.e. the full range of f/u was even wider  🡺 f/u of only 2 months in some is a little short for all-cause mortality |  | N |  |
| 8. For exposures that can vary in amount or level, did the study examine **different levels of the exposure** as related to the outcome (e.g., categories of exposure, or exposure measured as continuous variable)? |  | N |  |
| 9. Were the **exposure measures** (independent variables) clearly defined, valid, reliable, and implemented consistently across all study participants? | Y |  |  |
| 10. Was the **exposure(s) assessed more than once** over time? |  | N |  |
| 11. Were the **outcome measures** (dependent variables) clearly defined, valid, reliable, and implemented consistently across all study participants?  f/u by direct contact with pts or their physicians at the hospital/ OPD, TC with pt or family, and/or mail. | Y |  |  |
| 12. Were the **outcome assessors blinded** to the exposure status of participants? |  |  | NR |
| 13. Was **loss to follow-up** **after baseline 20% or less?**   - search for lost/ loss/ follow – nil. - 25% were excluded for ACS/ < 64 yo/ missing GNRI data 🡺 if only they’d told us how many had missing GNRI data, we could’ve calculated loss to f/u. no statement re. lost to f/u for mortality data. |  |  | CD |
| 14. Were key potential **confounding variables** measured and **adjusted statistically** for their impact on the relationship between exposure(s) and outcome(s)?  Multivariate analysis | Y |  |  |

*CD, cannot determine; NA, not applicable; NR, not reported

| Quality rating (Good/ Fair/ Poor) | Fair |
| --- | --- |
| Rater 1 initials: | Good |
| Rater 2 initials: | Fair |
| Additional comments, if Poor – why? | 1. 25% excluded for ACS/ < 64 yo/ missing GNRI. I wish they had specified ACS/ < 64 yo, which are genuine exclusion criteria, vs. missing GNRI which is loss to f/u. No statement re. lost to f/u for mortality data. Otherwise, it could have been ‘good’ 2. median f/u 189 days, IQR 66-421 days – i.e. some patients rather shorter f/u time. |

## 18. Kawakubo 2022 [West Tokyo Heart Failure registry == WET-HF]

|  | Yes | No | Other (CD, NR, NA) |
| --- | --- | --- | --- |
| 1. Was the **research question** or objective in this paper clearly stated?  End of intro: evaluated the prevalence of malnutrition among HFrEF patients, their long-term outcomes, and whether their nutritional status was associated with the use of combination medical therapy. | Y |  |  |
| 2. Was the **study population** clearly specified and defined?  Metods, Study design | Y |  |  |
| 3. Was the **participation rate** of eligible persons at least 50%?  Figure 1: HF pts =< 40%  Methods, patient population:  1713 consecutive pts hospitalised for HF  Exclusion: 73 died in hospital, 51 dialysis pts  == 1589 patients == base population  Lost to f/u: 124 lost to f/u, 229 missing albumin/ BMI, 5 missing data re. meds at d/c.  == 358 lost to f/u == 22.5%  Total analysed 1231. | Y |  |  |
| 4. Were **all the subjects selected or recruited from the same or similar populations** (including the same time period)? Were inclusion and exclusion criteria for being in the study prespecified and applied uniformly to all participants?  All tertiary care hospitals. | Y |  |  |
| 5. Was a **sample size** justification, power description, or variance and effect estimates provided?  🡺 Search for sample/ size/ power/calcul*/variance/estimate - nil |  | N |  |
| 6. For the analyses in this paper, were the **exposure**(s) of interest **measured prior to the outcome**(s) being measured? | Y |  |  |
| 7. Was the **timeframe sufficient** so that one could reasonably expect to see an association between exposure and outcome if it existed?  Results: GNRI and outcomes: median f/u 2 years [IQR 0.1-3.1 years] | Y |  |  |
| 8. For exposures that can vary in amount or level, did the study examine **different levels of the exposure** as related to the outcome (e.g., categories of exposure, or exposure measured as continuous variable)? | Y |  |  |
| 9. Were the **exposure measures** (independent variables) clearly defined, valid, reliable, and implemented consistently across all study participants? | Y |  |  |
| 10. Was the **exposure(s) assessed more than once** over time?  GNRI was calculated at d/c |  | N |  |
| 11. Were the **outcome measures** (dependent variables) clearly defined, valid, reliable, and implemented consistently across all study participants?  Search for ‘blind’ - nil |  |  | NR |
| 12. Were the **outcome assessors blinded** to the exposure status of participants?  Good | Y |  |  |
| 13. Was **loss to follow-up** **after baseline 20% or less?**  See above under participation rate .. total missing data & loss to f/u == 22.5% |  | N |  |
| 14. Were key potential **confounding variables** measured and **adjusted statistically** for their impact on the relationship between exposure(s) and outcome(s)? | Y |  |  |

*CD, cannot determine; NA, not applicable; NR, not reported

| Quality rating (Good/ Fair/ Poor) | Good |
| --- | --- |
| Rater 1 initials: | Good |
| Rater 2 initials: | Fair |
| Additional comments, if Poor – why? | - Good: GNRI calculated at time of d/c – i.e. when fluid overload [increasing BMI and haemodiluting albumin] has been brought down. - OK here: for f/u time - for some patients f/u was shorter [IQR 0.8 year == 9.6 months to 3.1 years] – but on average/ median it was long enough [2 years] – [cf. Honda 2016: median f/u 189 days, IQR 66-421 days] .. so OK for this one. |

## 19. Kinugasa 2013 [Tottori University Hospital, Yonago, Japan]

|  | Yes | No | Other (CD, NR, NA) |
| --- | --- | --- | --- |
| 1. Was the **research question** or objective in this paper clearly stated?  Stated in abstract [background] & at end of Introd. “Clinical significance of GNRI” – but for what? From outcome measures of all-cause mortality and HF re-admission this becomes clearer. | Y |  |  |
| 2. Was the **study population** clearly specified and defined?  Consecutive pts hospitalized with HFpEF Tottori Univ Hosp, Jan 2004 – April 2011. | Y |  |  |
| 3. Was the **participation rate** of eligible persons at least 50%?  Methods:  **Enrolled:** 194 consecutive pts, primary Dx HFpEF  **Exclusion crit:** HF etiology: severe valve disease, congenital disease, complete AV block, pericardial disease, primary pulmonary HTN, pulm. artery embolism, acute MI.  Exclusions from the 194 who met initial criteria: cancer [n=18], liver cirrhosis [n=2], on dialysis [n=1]. => 173 left  Exclusions for missing data: 21 pts = 12.1% [lack of body weight or lab data]  Analysed: 152 | Y |  |  |
| 4. Were **all the subjects selected or recruited from the same or similar populations** (including the same time period)? Were inclusion and exclusion criteria for being in the study prespecified and applied uniformly to all participants? | Y |  |  |
| 5. Was a **sample size** justification, power description, or variance and effect estimates provided?  🡺 Search for sample/ size/ power/calcul*/variance/estimate – nil. Sample size only mentioned in limitations. |  | N |  |
| 6. For the analyses in this paper, were the **exposure**(s) of interest **measured prior to the outcome**(s) being measured? | Y |  |  |
| 7. Was the **timeframe sufficient** so that one could reasonably expect to see an association between exposure and outcome if it existed?  P. 3/7 top: F/u median 2.1 years after hospital admission; IQR 1.2-3.6 years. | Y |  |  |
| 8. For exposures that can vary in amount or level, did the study examine **different levels of the exposure** as related to the outcome (e.g., categories of exposure, or exposure measured as continuous variable)? | Y |  |  |
| 9. Were the **exposure measures** (independent variables) clearly defined, valid, reliable, and implemented consistently across all study participants? | Y |  |  |
| 10. Was the **exposure(s) assessed more than once** over time? |  | N |  |
| 11. Were the **outcome measures** (dependent variables) clearly defined, valid, reliable, and implemented consistently across all study participants?  P. 3/7 top: F/u data obtained from medical records or telephone interview. | Y |  |  |
| 12. Were the **outcome assessors blinded** to the exposure status of participants? |  |  | NR |
| 13. Was **loss to follow-up** **after baseline 20% or less?**  See above: missing data/ lost to f/u 21 out of original 173 == 12.2%  No statement of none or how many lost to f/u. | Y |  |  |
| 14. Were key potential **confounding variables** measured and **adjusted statistically** for their impact on the relationship between exposure(s) and outcome(s)? | Y |  |  |

*CD, cannot determine; NA, not applicable; NR, not reported

| Quality rating (Good/ Fair/ Poor) | Fair |
| --- | --- |
| Rater 1 initials: | Fair |
| Rater 2 initials: | Poor |
| Additional comments, if Poor – why? | They seem to have done things right – except not stating whether any were lost to f/u. If they had stated this, it would have been good. |

## 20. Kitamura 2019 [Shinyukuhashi Hospital, Yukuhashi, Japan

|  | Yes | No | Other (CD, NR, NA) |
| --- | --- | --- | --- |
| 1. Was the **research question** or objective in this paper clearly stated?  Abstract: examine the effects of differences in nutritional status on ADL and mobility recovery of hospitalised elderly pts with HF. | Y |  |  |
| 2. Was the **study population** clearly specified and defined?  Fig 1 [p. 3/7]: of 377 consecutive HF rehab patients, 109 met inclusion criteria, 13 excluded [9 pacemaker surgery during admission, 2 change to other dptm, 1 died in hospital, 1 unmeasured ADL] 🡺 96 in study. | Y |  |  |
| 3. Was the **participation rate** of eligible persons at least 50%?  377 consecutive HF pts undergoing rehab  Inclusion crit: those who were age >= 65, could walk with assistance before admission, and admitted for first time included. => 109 left.  Exclusion crit: pacemaker surgery during admission, change to other deptm [n=2], died during admission n=12. => 97 left.  Missing data ADL n=1, change to other dpt n=2 🡺 3% missing data. | Y |  |  |
| 4. Were **all the subjects selected or recruited from the same or similar populations** (including the same time period)? Were inclusion and exclusion criteria for being in the study prespecified and applied uniformly to all participants? | Y |  |  |
| 5. Was a **sample size** justification, power description, or variance and effect estimates provided?  🡺 Search for sample/ size/ power/calcul*/variance/estimate – nil. |  | N |  |
| 6. For the analyses in this paper, were the **exposure**(s) of interest **measured prior to the outcome**(s) being measured? |  |  | NR |
| 7. Was the **timeframe sufficient** so that one could reasonably expect to see an association between exposure and outcome if it existed? | Y |  |  |
| 8. For exposures that can vary in amount or level, did the study examine **different levels of the exposure** as related to the outcome (e.g., categories of exposure, or exposure measured as continuous variable)? |  | N |  |
| 9. Were the **exposure measures** (independent variables) clearly defined, valid, reliable, and implemented consistently across all study participants? | Y |  |  |
| 10. Was the **exposure(s) assessed more than once** over time? |  | N |  |
| 11. Were the **outcome measures** (dependent variables) clearly defined, valid, reliable, and implemented consistently across all study participants?  ADL == motor FIM  Rivermead Mobility Index [RMI] – 14 questons  Performed twice [on admission and at d/c] by two physios..   - but together or independent and blinded ..   ? admission assessment about usual mobility, or mobility at moment of admission [i.e. skewed by the recent episode of HF]  ? always the same two physios or different ones | Y |  |  |
| 12. Were the **outcome assessors blinded** to the exposure status of participants? |  |  | NR |
| 13. Was **loss to follow-up** **after baseline 20% or less?**  Results, patient flow, and Fig 1: Missing data ADL n=1, change to other dpt n=2 🡺 3% missing data. | Y |  |  |
| 14. Were key potential **confounding variables** measured and **adjusted statistically** for their impact on the relationship between exposure(s) and outcome(s)? | Y |  |  |

*CD, cannot determine; NA, not applicable; NR, not reported

| Quality rating (Good/ Fair/ Poor) | Good |
| --- | --- |
| Rater 1 initials: | Fair |
| Rater 2 initials: | Good |
| Additional comments, if Poor – why? | They’ve done all the right things. they could have improved reporting by commenting on blinded assessment, two physios independently of e/other, how differences were resolved. |

## 21. Nishi 2019 [registry involves 11 hospitals in the Ibaraki prefecture, Japan -> Ibaraki Cardiovascular Assessment Study-HF registry .. 11 hospitals in Ibaraki prefecture]

|  | Yes | No | Other (CD, NR, NA) |
| --- | --- | --- | --- |
| 1. Was the **research question** or objective in this paper clearly stated?  🡺 Yes: whether GNRI at d/c may be helpful in predicting the long-term. prognosis of patients hospitalized with HFpEF, LVEF >= 50% | Y |  |  |
| 2. Was the **study population** clearly specified and defined?  >= 65-yo with HFpEF on the registry | Y |  |  |
| 3. Was the **participation rate** of eligible persons at least 50%?  Fig 1:  Registry: 838 patients informed consent  Total 442 excluded from the whole group:   - 196 < 65 yo - 7 on dialysis - 25 in-hospital deaths [GNRI at d/c was their independent variable] 🡺838 – 228 = 610 left. - 27 transferred elsewhere for continued med care [== lost to f/u to my mind] - 187 pts missing GNRF 🡺   🡺 187 missing GNRI + 27 transferred elsewhere, out of 610 after genuine exclusions == 214/610 == 35% lost to f/u.  🡺 396 left, of whom 286 HFrEF [therefore excluded from this study], 110 analysed as HFpEF. | Y |  |  |
| 4. Were **all the subjects selected or recruited from the same or similar populations** (including the same time period)? Were inclusion and exclusion criteria for being in the study prespecified and applied uniformly to all participants?  Hospitalized or HF, Ibaraki prefecture, June 2012-March 2015. | Y |  |  |
| 5. Was a **sample size** justification, power description, or variance and effect estimates provided?  🡺 Search for sample/ size/ power/calcul*/variance/estimate - nil |  | N |  |
| 6. For the analyses in this paper, were the **exposure**(s) of interest **measured prior to the outcome**(s) being measured? | Y |  |  |
| 7. Was the **timeframe sufficient** so that one could reasonably expect to see an association between exposure and outcome if it existed?  Pts enrolled in registry June 2012-March 2015.  F/u til 31 March 2016. | Y |  |  |
| 8. For exposures that can vary in amount or level, did the study examine **different levels of the exposure** as related to the outcome (e.g., categories of exposure, or exposure measured as continuous variable)? |  | N |  |
| 9. Were the **exposure measures** (independent variables) clearly defined, valid, reliable, and implemented consistently across all study participants?  Although they could have defined ‘at discharge’ more specifically – in the last 24/48 hours before d/c? | Y |  |  |
| 10. Was the **exposure(s) assessed more than once** over time? | N |  |  |
| 11. Were the **outcome measures** (dependent variables) clearly defined, valid, reliable, and implemented consistently across all study participants?  Not reported how they assessed survival status – whether only in hospital computers or also from other medical facilities / family. | N |  |  |
| 12. Were the **outcome assessors blinded** to the exposure status of participants? |  |  | NR |
| 13. Was **loss to follow-up** **after baseline 20% or less?**  See Q3. From the whole group [HFrEF + HFpEF] 35% were excluded for missing GNRI data/ transfer elsewhere for med. care.  No statement about further loss to f/u of patients not traceable. |  | N |  |
| 14. Were key potential **confounding variables** measured and **adjusted statistically** for their impact on the relationship between exposure(s) and outcome(s)?  Adjusted for age and sex [model 2] and for logBNP [model 3] .. so less than some other studies. Probably no adjustment for LVEF because this study looked only at pple with preserved EF. | Y |  |  |

*CD, cannot determine; NA, not applicable; NR, not reported

| Quality rating (Good/ Fair/ Poor) | Poor |
| --- | --- |
| Rater 1 initials: | Fair |
| Rater 2 initials: | Poor |
| Additional comments, if Poor – why? | [1] 35% loss to f/u because missing data/ transferred elsewhere for further med. care.  [2] No statement about loss to f/u – i.e. for whom no info about alive or deceased, and/or no info on cause of death.  [3] I find fig. 1 not logical. I would have liked to see: 838 patients .. X genuine exclusions [dialysis, in-hospital death] -> of those left Y had HFpEF -> of those Z excluded for missing data/ transferred elsewhere for further care = % lost to f/u. |

## 22. Sargento 2017 [University Hospital in Portugal]

|  | Yes | No | Other (CD, NR, NA) |
| --- | --- | --- | --- |
| 1. Was the **research question** or objective in this paper clearly stated? | Y |  |  |
| 2. Was the **study population** clearly specified and defined?  **Methods**, study population  Of 268 patients with HFrEF followed up in HF clinic  **178 were > 65-yo, 143 met incl. crit. of systolic HF.**  All clinically stable, optimal Tx, nurse-led education program completed.  Of 178 eligible patients, n=25 [20%] were rejected because of missing albumin values.  25/178 = 14%, not 20%.  25/143 = 17.5%  🡺 was 25 a typo? if n=35/178 excluded for missing albumin values, that makes 19.7%, and results in 143 patients studied.  **Abstract: total of 143 were studied.** | Y |  |  |
| 3. Was the **participation rate** of eligible persons at least 50%? | Y |  |  |
| 4. Were **all the subjects selected or recruited from the same or similar populations** (including the same time period)? Were inclusion and exclusion criteria for being in the study prespecified and applied uniformly to all participants? | Y |  |  |
| 5. Was a **sample size** justification, power description, or variance and effect estimates provided?  🡺 Search for sample/ size/ power/calcul*/variance/estimate |  | N |  |
| 6. For the analyses in this paper, were the **exposure**(s) of interest **measured prior to the outcome**(s) being measured? | Y |  |  |
| 7. Was the **timeframe sufficient** so that one could reasonably expect to see an association between exposure and outcome if it existed?  Methods: f/u time 3 years, until 1 May 2016.  Enrolment > 2000 at community HF clinic.  Results: median f/u 1683 days [IQR 1096-2230] | Y |  |  |
| 8. For exposures that can vary in amount or level, did the study examine **different levels of the exposure** as related to the outcome (e.g., categories of exposure, or exposure measured as continuous variable)?  They compared GNRI tertiles [Methods, stats] | Y |  |  |
| 9. Were the **exposure measures** (independent variables) clearly defined, valid, reliable, and implemented consistently across all study participants?  Measured “at the baseline visit” [Methods, baseline characteristics] | Y |  |  |
| 10. Was the **exposure(s) assessed more than once** over time? |  | N |  |
| 11. Were the **outcome measures** (dependent variables) clearly defined, valid, reliable, and implemented consistently across all study participants?  They phoned relatives if pt did not attend for f/u. | Y |  |  |
| 12. Were the **outcome assessors blinded** to the exposure status of participants? |  |  | NR |
| 13. Was **loss to follow-up** **after baseline 20% or less?**  Patients who did not attend appointments were f/u by phone. | Y |  |  |
| 14. Were key potential **confounding variables** measured and **adjusted statistically** for their impact on the relationship between exposure(s) and outcome(s)? | Y |  |  |

*CD, cannot determine; NA, not applicable; NR, not reported

| Quality rating (Good/ Fair/ Poor) | Good |
| --- | --- |
| Rater 1 initials: | Fair |
| Rater 2 initials: | Good |
| Additional comments, if Poor – why? | Apart from the typo [not 25 but 35 excluded for missing values], they’ve done all the right things.  No statement whether they managed to follow up each and every one of the patients, but I take their efforts to phone patients in lieu of that. |

## 23. Sze 2019 [Hull, Yorkshire]

|  | Yes | No | Other (CD, NR, NA) |
| --- | --- | --- | --- |
| 1. Was the **research question** or objective in this paper clearly stated?  Relation between congestion, malnutrition and mortality. | Y |  |  |
| 2. Was the **study population** clearly specified and defined?  Community CHF clinic, enrolled 2008-2012 “for whom we have detailed echocardiographic images” | Y |  |  |
| 3. Was the **participation rate** of eligible persons at least 50%?  They do not indicate how many were excluded for missing data. |  |  | NR |
| 4. Were **all the subjects selected or recruited from the same or similar populations** (including the same time period)? Were inclusion and exclusion criteria for being in the study prespecified and applied uniformly to all participants? | Y |  |  |
| 5. Was a **sample size** justification, power description, or variance and effect estimates provided?  🡺 Search for sample/ size/ power/calcul*/variance/estimate - nil |  | N |  |
| 6. For the analyses in this paper, were the **exposure**(s) of interest **measured prior to the outcome**(s) being measured?  At index visit. | Y |  |  |
| 7. Was the **timeframe sufficient** so that one could reasonably expect to see an association between exposure and outcome if it existed?  median f/u 1683 days [IQR: 1096-2230] | Y |  |  |
| 8. For exposures that can vary in amount or level, did the study examine **different levels of the exposure** as related to the outcome (e.g., categories of exposure, or exposure measured as continuous variable)? | Y |  |  |
| 9. Were the **exposure measures** (independent variables) clearly defined, valid, reliable, and implemented consistently across all study participants?  Not reported whether they were all done at the point of referral to the clinic – one assumes so. |  |  | NR |
| 10. Was the **exposure(s) assessed more than once** over time? |  | N |  |
| 11. Were the **outcome measures** (dependent variables) clearly defined, valid, reliable, and implemented consistently across all study participants?  Pts consented to access to all primary and secondary care records – and in UK that means accurate f/u. | Y |  |  |
| 12. Were the **outcome assessors blinded** to the exposure status of participants? |  |  | NR |
| 13. Was **loss to follow-up** **after baseline 20% or less?**  They only included patients for whom they had detailed echo data. No mention of how many were excluded.  P. 2/10 bottom left “Outcome was censored at the point of last medical contact in primary or secondary care.” As they had consent to access all 1ary and 2ary care records, that is as good f/u as you can get. |  |  | CD |
| 14. Were key potential **confounding variables** measured and **adjusted statistically** for their impact on the relationship between exposure(s) and outcome(s)? | Y |  |  |

*CD, cannot determine; NA, not applicable; NR, not reported

| Quality rating (Good/ Fair/ Poor) | Fair |
| --- | --- |
| Rater 1 initials: | Fair |
| Rater 2 initials: | Poor |
| Additional comments, if Poor – why? | Only included patients for whom they had echo data. No indication how many were excluded for unavailable data. |

## 24. Yasumura 2020

|  | Yes | No | Other (CD, NR, NA) |
| --- | --- | --- | --- |
| 1. Was the **research question** or objective in this paper clearly stated? | Y |  |  |
| 2. Was the **study population** clearly specified and defined?  203 consecutive pts with ADHF at National Hospital Organization Osaka National Hospital April 2015-Jan 2017. | Y |  |  |
| 3. Was the **participation rate** of eligible persons at least 50%?  Excluded: 26 pts whose condition was serious – all 26 died during hospitalization [this is common in these studies].  Also excluded: when GNRI could not be calculated or missing data. 🡺 They do not say how many people were excluded for missing data. |  |  | CD |
| 4. Were **all the subjects selected or recruited from the same or similar populations** (including the same time period)? Were inclusion and exclusion criteria for being in the study prespecified and applied uniformly to all participants? | Y |  |  |
| 5. Was a **sample size** justification, power description, or variance and effect estimates provided?  🡺 Search for sample/ size/ power/calcul*/variance/estimate  Sample size only comes in Limitations. |  | N |  |
| 6. For the analyses in this paper, were the **exposure**(s) of interest **measured prior to the outcome**(s) being measured? | Y |  |  |
| 7. Was the **timeframe sufficient** so that one could reasonably expect to see an association between exposure and outcome if it existed? | Y |  |  |
| 8. For exposures that can vary in amount or level, did the study examine **different levels of the exposure** as related to the outcome (e.g., categories of exposure, or exposure measured as continuous variable)? | Y |  |  |
| 9. Were the **exposure measures** (independent variables) clearly defined, valid, reliable, and implemented consistently across all study participants?  GNRI: yes .. but not when this was done – adm or d/c  SWT: simple walking test at d/c: < 200 m = reduced physical capacity.  Lab: at admission and at d/c  Echo: at d/c  Simple walking test SWT: during rehab .. different levels [stepping exercise, in room, 50, 100, 200 m, bicycle ergometer – patient passed a level when they could do it without resting, Borg 11-13 [light – somewhat hard], and without critical changes in vital signs => would move up to the next level.  BNP – measured at d/c [p 3/8 2^nd^ para ri] | Y |  |  |
| 10. Was the **exposure(s) assessed more than once** over time?  GNRI: not reported whether at adm or on d/c |  |  | NR |
| 11. Were the **outcome measures** (dependent variables) clearly defined, valid, reliable, and implemented consistently across all study participants?  Nothing in methods about outcomes or how they were assessed. |  |  | NR |
| 12. Were the **outcome assessors blinded** to the exposure status of participants? |  |  | NR |
| 13. Was **loss to follow-up** **after baseline 20% or less?**  203 patients enrolled  excluded re. missing data ? how many  20 pts lost to f/u < 2 years from d/c |  |  | CD |
| 14. Were key potential **confounding variables** measured and **adjusted statistically** for their impact on the relationship between exposure(s) and outcome(s)?  P. 3/8 bottom right, in Table 3: adjusted for the combined index, age, BNP, Hb, CRP. | Y |  |  |

*CD, cannot determine; NA, not applicable; NR, not reported

| Quality rating (Good/ Fair/ Poor) | Fair |
| --- | --- |
| Rater 1 initials: | Good |
| Rater 2 initials: | Poor |
| Additional comments, if Poor – why? | They don’t say how many they excluded in the beginning for missing data – therefore cannot determine the accuracy of the study. |

**Certainty of the evidence**

| **Table S4.** Summary of findings for comparisons using GNRI score. | | | | | | |
| --- | --- | --- | --- | --- | --- | --- |
| **Abnormal GNRI score compared to normal GNRI score for patients with heart failure** | | | | | | |
| **Patient or population: P**atients with heart failure  **Exposure:** abnormal GNRI score  **Comparison:** normal GNRI score | | | | | | |
| Outcomes | **Anticipated absolute effects^*^** (95% CI) | | Relative effect (95% CI) | № of participants (studies) | Certainty of the evidence (GRADE) | Comments |
|  | **Risk with normal GNRI score** | **Risk with abnormal GNRI score** |  |  |  |  |
| BNP levels | The mean BNP levels was **454** pg/mL | MD **205 pg/mL higher** (101 higher to 309 higher) | - | 3373 (8 non-randomised studies) | ⨁⨁◯◯ Low | Patients with abnormal GNRI scores may have higher BNP levels. |
| NT-proBNP levels | The mean nT-proBNP levels was **1,053** pg/mL | MD **1885 pg/mL higher** (1,429 higher to 2,342 higher) | - | 1095 (2 non-randomised studies) | ⨁⨁⨁◯ Moderate^a^ | Patients with abnormal GNRI scores likely have higher NT-proBNP levels. |
| CRP levels | The mean CRP levels was **0.91** mg/dL | MD **0.50 mg/dL higher** (0.12 higher to 0.88 higher) | - | 1156 (5 non-randomised studies) | ⨁⨁◯◯ Low | Patients with abnormal GNRI scores may have higher CRP levels. |
| ***The risk in the intervention group** (and its 95% confidence interval) is based on the assumed risk in the comparison group and the **relative effect** of the intervention (and its 95% CI).  **CI:** confidence interval; **MD:** mean difference | | | | | | |
| **GRADE Working Group grades of evidence** **High certainty:** we are very confident that the true effect lies close to that of the estimate of the effect. **Moderate certainty:** we are moderately confident in the effect estimate: the true effect is likely to be close to the estimate of the effect, but there is a possibility that it is substantially different. **Low certainty:** our confidence in the effect estimate is limited: the true effect may be substantially different from the estimate of the effect. **Very low certainty:** we have very little confidence in the effect estimate: the true effect is likely to be substantially different from the estimate of effect. | | | | | | |

#### Explanations

a. Upgraded for large effect size: NT-proBNP levels much higher among patients with abnormal GNRI score

| **Table S5.** Summary of findings for comparisons using CONUT score. | | | | | | |
| --- | --- | --- | --- | --- | --- | --- |
| **Abnormal CONUT score compared to normal CONUT score for patients with heart failure** | | | | | | |
| **Patient or population: P**atients with heart failure  **Exposure:** abnormal CONUT score  **Comparison:** normal CONUT score | | | | | | |
| Outcomes | **Anticipated absolute effects^*^** (95% CI) | | Relative effect (95% CI) | № of participants (studies) | Certainty of the evidence (GRADE) | Comments |
|  | **Risk with normal CONUT score** | **Risk with abnormal CONUT score** |  |  |  |  |
| BNP levels | The mean BNP levels was **418** pg/mL | MD **159 pg/mL higher** (2 lower to 319 higher) | - | 2613 (5 non-randomised studies) | ⨁◯◯◯ Very low^a^ | The evidence is very uncertain about the association of abnormal CONUT score with BNP levels. |
| NT-proBNP levels | The mean nT-proBNP levels was 2,660 pg/mL | MD **1,160 pg/mL higher** (701 higher to 1,619 higher) | - | 939 (5 non-randomised studies) | ⨁⨁◯◯ Low^b^ | Patients with abnormal GNRI scores may have higher NT-proBNP levels. |
| CRP levels | The mean CRP levels was **0.35** mg/dL | MD **0.4 mg/dL higher** (0.08 higher to 0.72 higher) | - | 2463 (4 non-randomised studies) | ⨁⨁◯◯ Low | Patients with abnormal CONUT scores may have higher CRP levels. |
| ***The risk in the intervention group** (and its 95% confidence interval) is based on the assumed risk in the comparison group and the **relative effect** of the intervention (and its 95% CI).  **CI:** confidence interval; **MD:** mean difference | | | | | | |
| **GRADE Working Group grades of evidence** **High certainty:** we are very confident that the true effect lies close to that of the estimate of the effect. **Moderate certainty:** we are moderately confident in the effect estimate: the true effect is likely to be close to the estimate of the effect, but there is a possibility that it is substantially different. **Low certainty:** our confidence in the effect estimate is limited: the true effect may be substantially different from the estimate of the effect. **Very low certainty:** we have very little confidence in the effect estimate: the true effect is likely to be substantially different from the estimate of effect. | | | | | | |

#### Explanations

a. Downgraded for imprecision: CI include no change or high clinically meaningful change

b. Most of the patients were enrolled in studies with high risk of bias, however, sensitivity analyses excluding these studies yielded similar results

**Meta-regression analysis**

**Table S6.** Meta-regression analyses to explore the impact of potential covariates.

| Outcome of interest (Malnutrition tool) Potential covariate | *r* | SE | 95% CI | *z* | *p* |
| --- | --- | --- | --- | --- | --- |
| BNP (GNRI) - Age | -0.531 | 6.9793 | -14.21 – 13.15 | -0.08 | 0.94 |
| BNP (GNRI) - LVEF | -0.1492 | 2.5677 | -5.18 – 4.88 | -0.06 | 0.95 |
| CRP (GNRI) - Age | -0.1238 | 0.0616 | -0.24 – -0.00 | -2.01 | 0.04* |
| CRP (GNRI) - LVEF | -0.0234 | 0.0276 | -0.08 – 0.03 | -0.85 | 0.40 |
| BNP (CONUT) - Age | 0.1645 | 7.4917 | -14.52 – 14.85 | 0.02 | 0.98 |
| BNP (CONUT) - LVEF | 0.0688 | 7.7561 | -15.13 – 15.27 | 0.01 | 0.99 |
| BNP (CONUT) - BMI | -0.0546 | 48.9733 | -96.04 – 95.93 | -0.00 | 0.99 |
| CRP (CONUT) - Age | 0.0555 | 0.0450 | -0.03 – 0.14 | 1.23 | 0.22 |
| CRP (CONUT) - LVEF | 0.0025 | 0.0912 | -0.18 – 0.18 | 0.03 | 0.98 |
| CRP (CONUT) - BMI | 0.0893 | 0.0842 | -0.08 – 0.25 | 1.06 | 0.29 |

BMI, body mass index; BNP, brain natriuretic peptide; CI, confidence interval; CONUT, Controlling Nutritional Status; GNRI, Geriatric Nutritional Risk Index; LVEF, left ventricular ejection fraction; SE, standard error.
*indicates significance (p < 0.05).

**Sensitivity analyses**

**
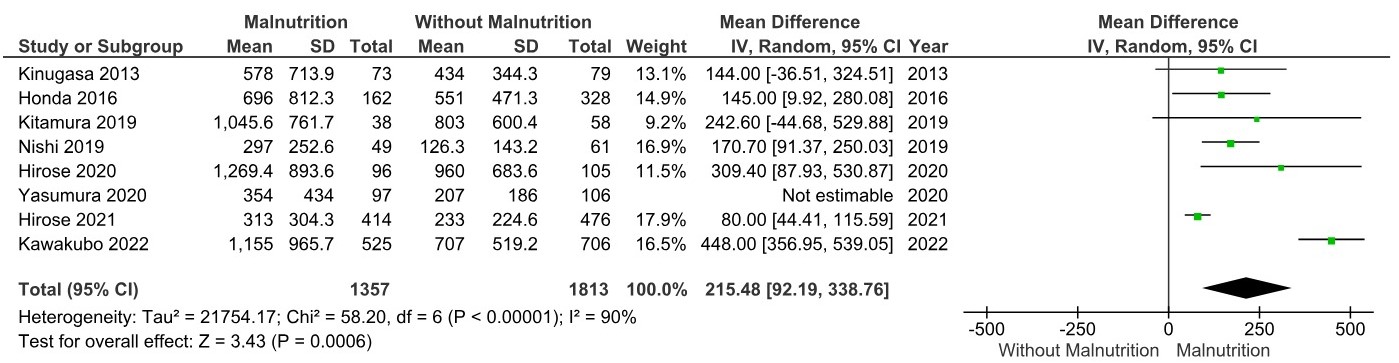
Figure S1.** Mean differences in BNP levels according to malnutrition status established with the use of GNRI in heart failure patients after exclusion of studies with higher rate of comorbid valvular disease in malnourished patients. Mean differences are presented as 95% confidence intervals using random effects model.

**
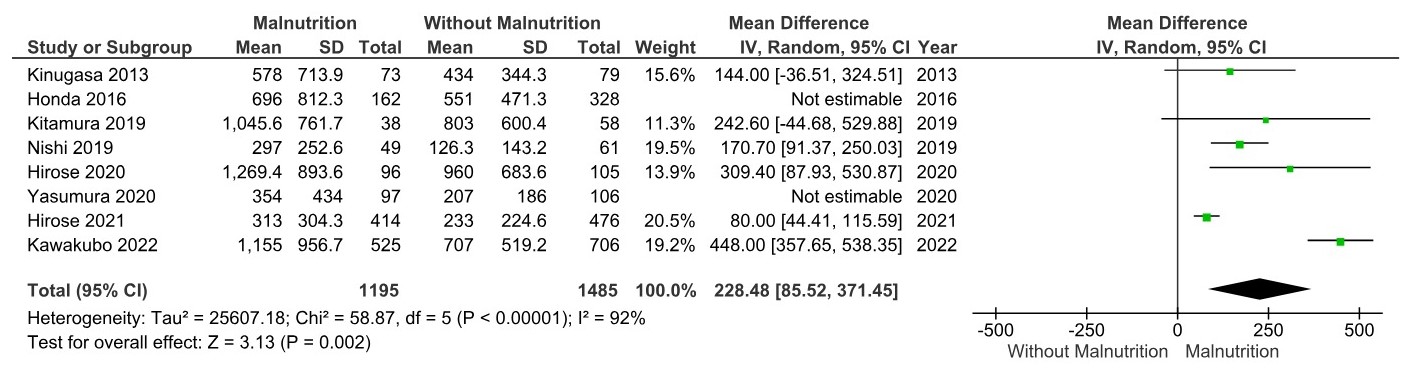
**

**Figure S2.** Mean differences in BNP levels according to malnutrition status established with the use of GNRI in heart failure patients after exclusion of studies with higher prevalence of valvular disease and prior HF admission in the malnourished group. Mean differences are presented as 95% confidence intervals using random effects model.

**
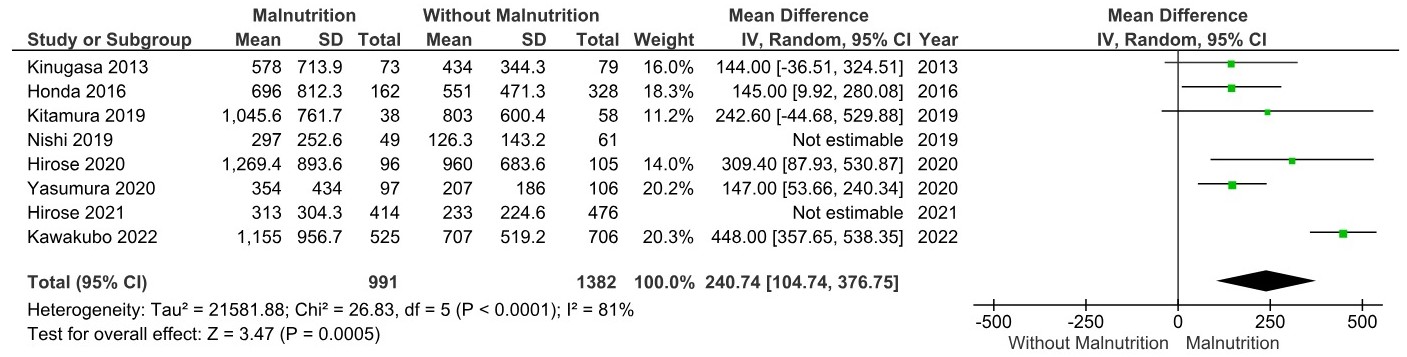
**

**Figure S3.** Mean differences in BNP levels according to malnutrition status established with the use of GNRI in heart failure patients after exclusion of studies with high risk of bias. Mean differences are presented as 95% confidence intervals using random effects model.

**
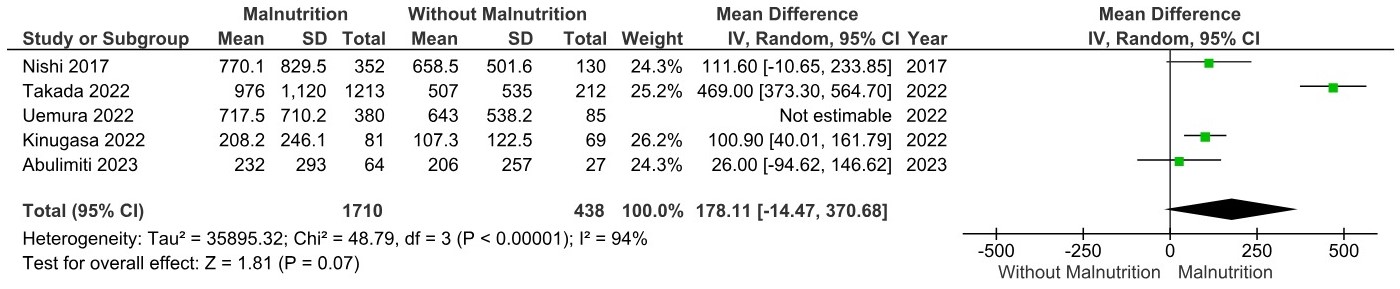
**

**Figure S4.** Mean differences in BNP levels according to malnutrition status established with the use of CONUT score in heart failure patients after exclusion of studies with higher prevalence of acute infection, malignancy, and frailty in the malnourished group. Mean differences are presented as 95% confidence intervals using random effects model.

**
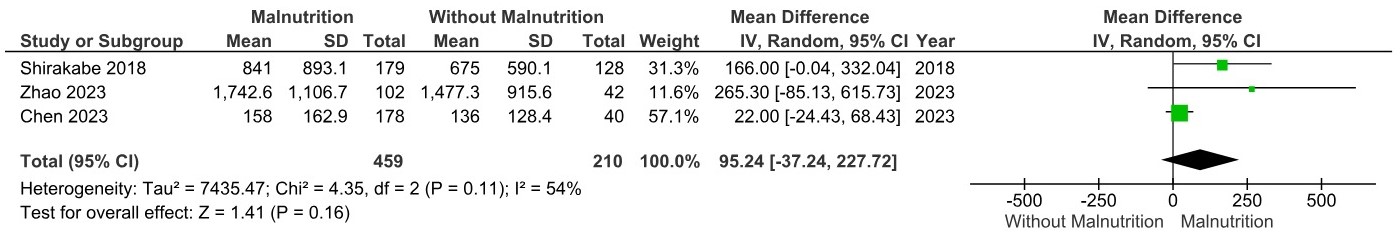
**

**Figure S5.** Mean differences in BNP levels in patients with mild malnutrition (CONUT scores of 2-4) compared to normal nutrition and heart failure. Mean differences are presented as 95% confidence intervals using random effects model.

**
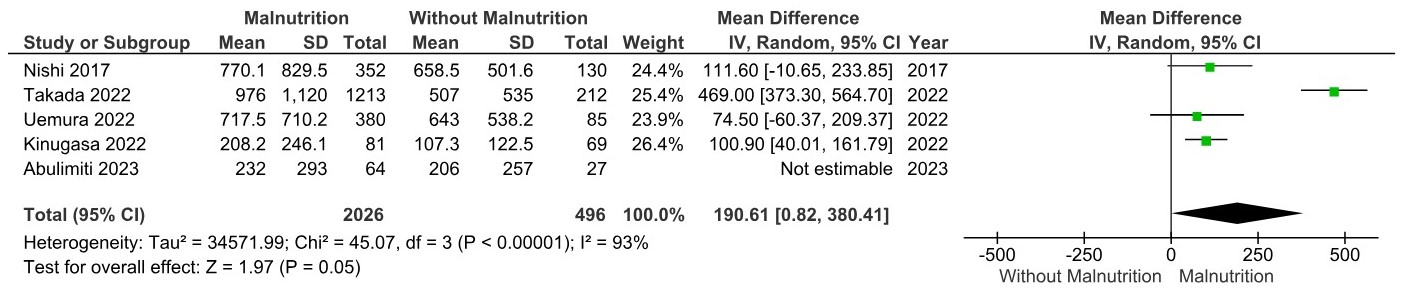
**

**Figure S6.** Mean differences in BNP levels according to malnutrition status established with the use of CONUT score in heart failure patients after exclusion of studies with high risk of bias. Mean differences are presented as 95% confidence intervals using random effects model.

**
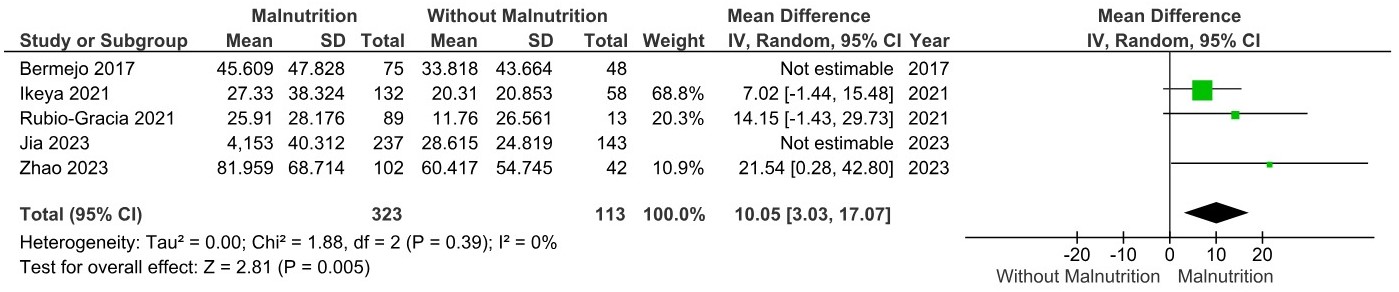
**

**Figure S7.** Mean differences in NT-proBNP levels according to malnutrition status established with the use of CONUT in heart failure patients after exclusion of studies with high risk of bias. Mean differences are presented as 95% confidence intervals using random effects model.

**
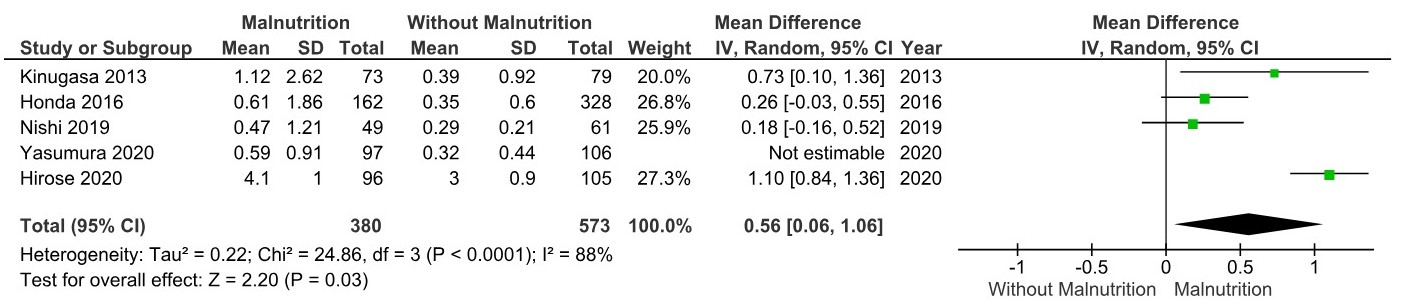
**

**Figure S8.** Mean differences in CRP levels according to malnutrition status established with the use of GNRI in heart failure patients after exclusion of studies with higher prevalence of valvular disease in the malnourished group. Mean differences are presented as 95% confidence intervals using random effects model.

**
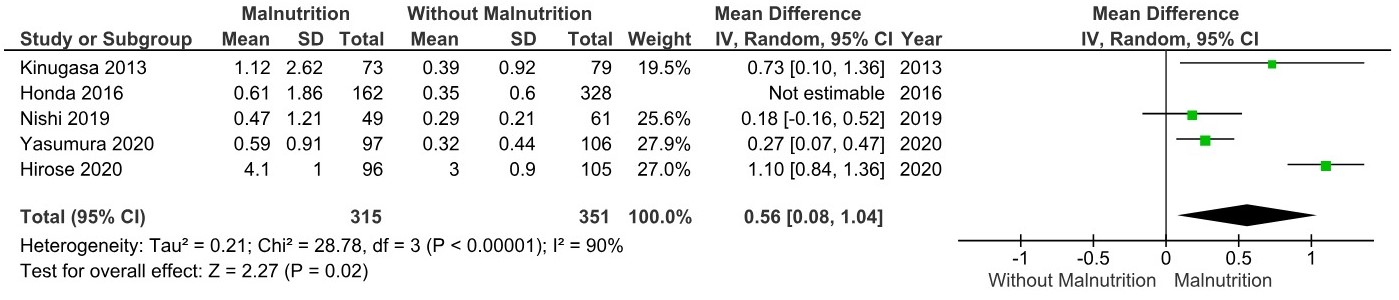
**

**Figure S9.** Mean differences in CRP levels according to malnutrition status established with the use of GNRI in heart failure patients after exclusion of studies with higher prevalence of prior HF admission in the malnourished group. Mean differences are presented as 95% confidence intervals using random effects model.

**
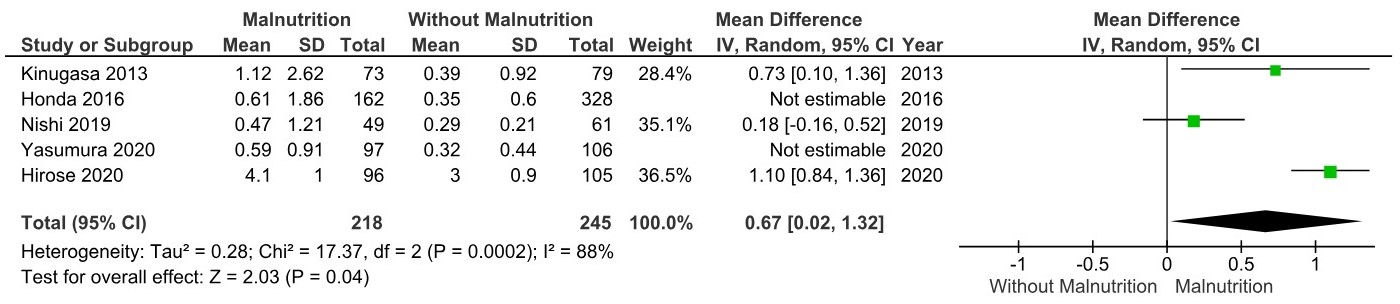
**

**Figure S10.** Mean differences in CRP levels according to malnutrition status established with the use of GNRI in heart failure patients after exclusion of studies with higher prevalence of valvular disease and prior HF admission in the malnourished group. Mean differences are presented as 95% confidence intervals using random effects model.

**
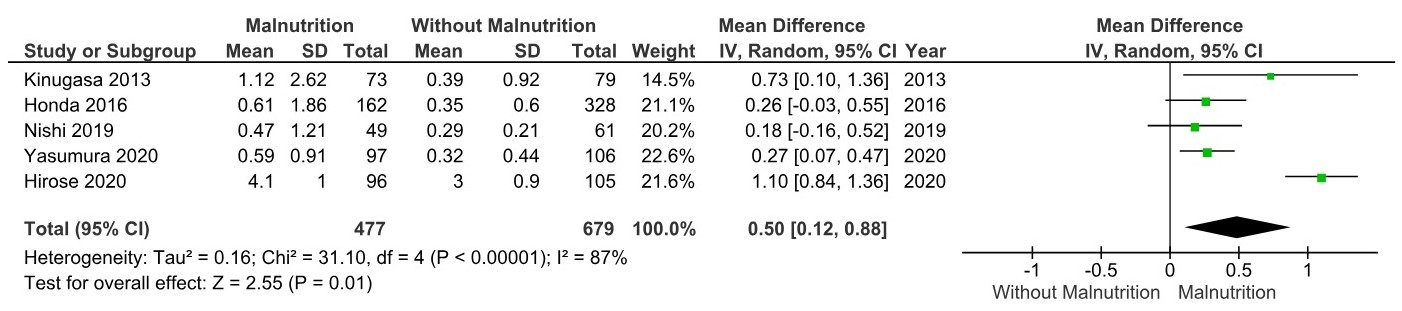
**

**Figure S11.** Mean differences in CRP levels according to malnutrition status established with the use of GNRI in heart failure patients after exclusion of studies with higher prevalence of hemodialysis in the malnourished group. Mean differences are presented as 95% confidence intervals using random effects model.

**
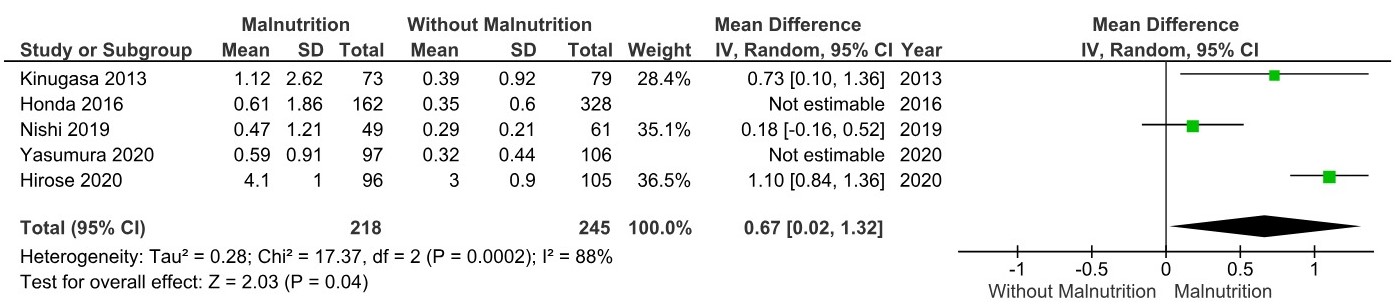
**

**Figure S12.** Mean differences in CRP levels according to malnutrition status established with the use of GNRI in heart failure patients after exclusion of studies with higher prevalence of hemodialysis, valvular disease, and previous hospitalization due to HF in the malnourished group. Mean differences are presented as 95% confidence intervals using random effects model.

**
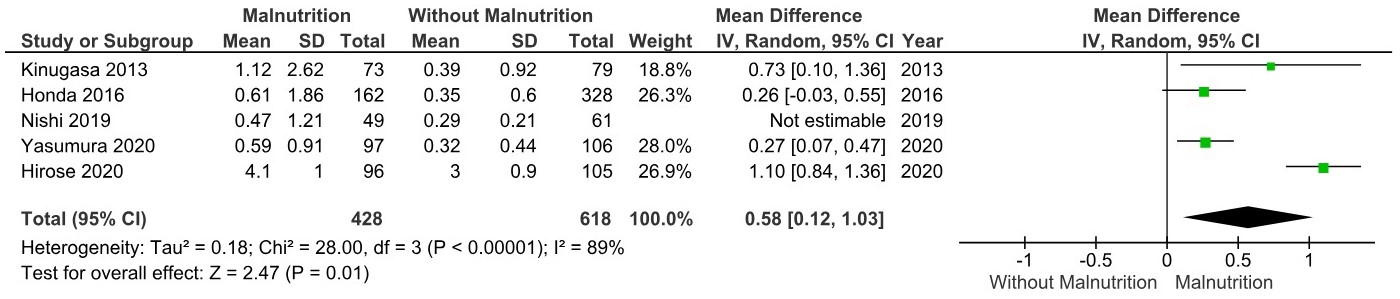
**

**Figure S13.** Mean differences in CRP levels according to malnutrition status established with the use of GNRI in heart failure patients after exclusion of one study with high risk of bias. Mean differences are presented as 95% confidence intervals using random effects model.

**
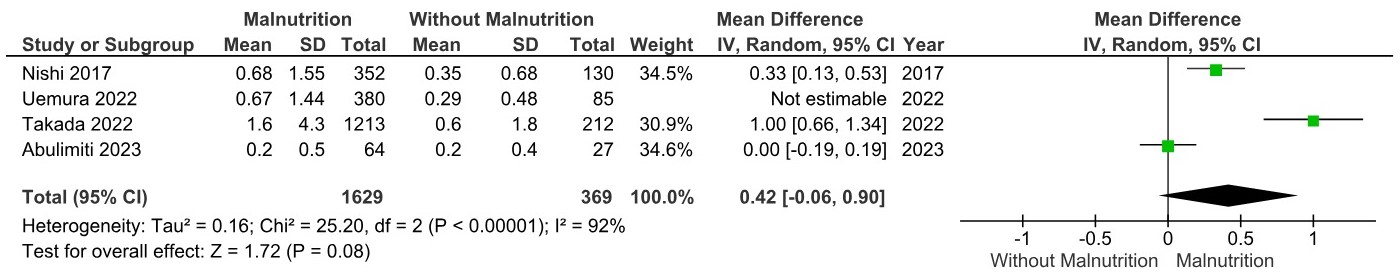
**

**Figure S14.** Mean differences in CRP levels according to malnutrition status established with the use of CONUT scores in heart failure patients after exclusion of studies with higher prevalence of acute infection, malignancy, and frailty in the malnourished group. Mean differences are presented as 95% confidence intervals using random effects model.

**
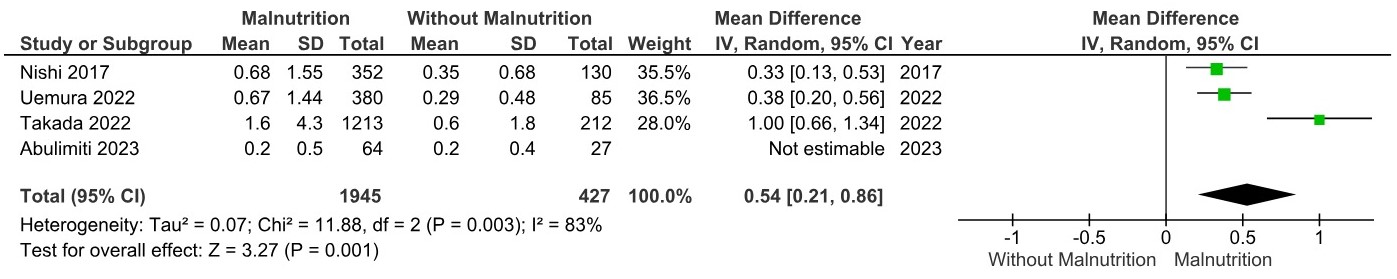
**

**Figure S15.** Mean differences in CRP levels according to malnutrition status established with the use of CONUT score in heart failure patients after exclusion of one study with high risk of bias. Mean differences are presented as 95% confidence intervals using random effects model.


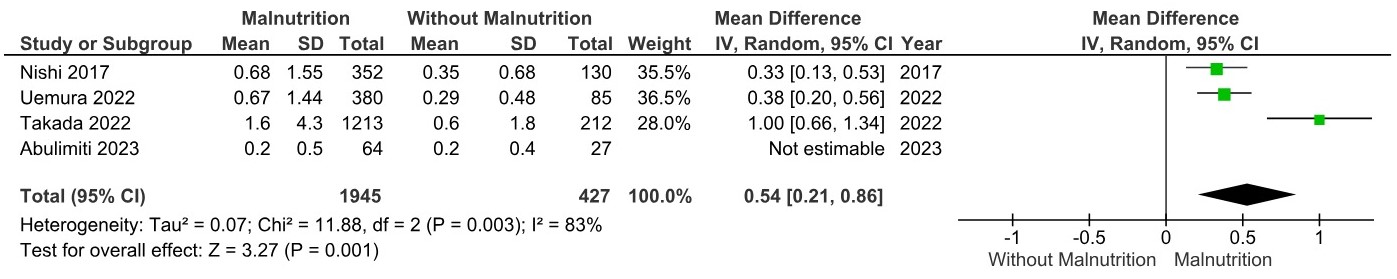

Supplement: Supplementary file 2 — Table S1. Search terms employed in the screening based on title, abstract, and keywords in the literature search. Table S2. Risk of bias assessment using CONUT scores. Table S3. Risk of bias assessment using GNRI scores. Table S4. Summary of findings for comparisons using GNRI score. Table S5. Summary of findings for comparisons using CONUT score. Table S6. Meta‐regression analyses to explore the impact of potential covariates. Figure S1. Mean differences in BNP levels according to malnutrition status established with the use of GNRI in heart failure patients after exclusion of studies with higher rate of comorbid valvular disease in malnourished patients. Mean differences are presented as 95% confidence intervals using random effects model. Figure S2. Mean differences in BNP levels according to malnutrition status established with the use of GNRI in heart failure patients after exclusion of studies with higher prevalence of valvular disease and prior HF admission in the malnourished group. Mean differences are presented as 95% confidence intervals using random effects model. Figure S3. Mean differences in BNP levels according to malnutrition status established with the use of GNRI in heart failure patients after exclusion of studies with high risk of bias. Mean differences are presented as 95% confidence intervals using random effects model. Figure S4. Mean differences in BNP levels according to malnutrition status established with the use of CONUT score in heart failure patients after exclusion of studies with higher prevalence of acute infection, malignancy, and frailty in the malnourished group. Mean differences are presented as 95% confidence intervals using random effects model. Figure S5. Mean differences in BNP levels in patients with mild malnutrition (CONUT scores of 2–4) compared to normal nutrition and heart failure. Mean differences are presented as 95% confidence intervals using random effects model. Figure S6. Mean differences in BNP levels according [file EHF2-11-3052-s001.docx]
